# Orally Administrated Hydrogel Harnessing Intratumoral Microbiome and Microbiota-Related Immune Responses for Potentiated Colorectal Cancer Treatment

**DOI:** 10.34133/research.0364

**Published:** 2024-05-08

**Authors:** Lei Li, Shouhua He, Boyi Liao, Manchun Wang, Huimin Lin, Ben Hu, Xinyue Lan, Zhilin Shu, Chao Zhang, Meng Yu, Zhaowei Zou

**Affiliations:** ^1^ Department of General Surgery, Zhujiang Hospital, Southern Medical University, Guangzhou 510282, China.; ^2^ NMPA Key Laboratory for Research and Evaluation of Drug Metabolism and Guangdong Provincial Key Laboratory of New Drug Screening and Guangdong-Hongkong-Macao Joint Laboratory for New Drug Screening, School of Pharmaceutical Sciences, Southern Medical University, Guangzhou 510515, China.

## Abstract

The intestinal and intratumoral microbiota are closely associated with tumor progression and response to antitumor treatments. The antibacterial or tumor microenvironment (TME)-modulating approaches have been shown to markedly improve antitumor efficacy, strategies focused on normalizing the microbial environment are rarely reported. Here, we reported the development of an orally administered inulin-based hydrogel with colon-targeting and retention effects, containing hollow MnO_2_ nanocarrier loaded with the chemotherapeutic drug Oxa (Oxa@HMI). On the one hand, beneficial bacteria in the colon specifically metabolized Oxa@HMI, resulting in the degradation of inulin and the generation of short-chain fatty acids (SCFAs). These SCFAs play a crucial role in modulating microbiota and stimulating immune responses. On the other hand, the hydrogel matrix underwent colon microbiota-specific degradation, enabling the targeted release of Oxa and production of reactive oxygen species in the acidic TME. In this study, we have established, for the first time, a microbiota-targeted drug delivery system Oxa@HMI that exhibited high efficiency in colorectal cancer targeting and colon retention. Oxa@HMI promoted chemotherapy efficiency and activated antitumor immune responses by intervening in the microbial environment within the tumor tissue, providing a crucial clinical approach for the treatment of colorectal cancer that susceptible to microbial invasion.

## Introduction

The symbiotic homeostasis of the intestinal microbiota, often referred to as the “forgotten organ”, plays an important role in host health. Abundant clinical data have shown that a diverse population of intestinal bacteria can enter the circulation through the mucosal system, accumulate and colonize in various types of tumor tissues, forming “intratumoral microbiota” that proliferates with tumor progression [1]. The intratumoral microbiota profoundly impacts tumor initiation, progression, and response to treatment. It exerts this influence through modulating oncogenic signaling pathways, promoting mutagenesis, altering the metabolism of chemotherapeutics, and shaping the host immune response [2,3]. On the one hand, tumor-promoting pathogenic bacteria within the intratumoral microbiota, such as *F. nucleatum*, *Veillonella*, *Prevotella*, and *Streptococcus*, have been identified to promote tumor progression and lead to drug resistance through inducing DNA damage [4], promoting inflammation [5], inducing tumor proliferation [6,7], and protecting tumors from immune attacks [8]. On the other hand, certain probiotic bacteria, such as *Lactobacillus*, *Bifidobacterium*, Lachnospiraceae, *Roseburia*, and *Eubacterium rectale*, which are depleted in colorectal cancer patients, have been found to produce short-chain fatty acids (SCFAs) as metabolic byproducts. These metabolites can activate tumor immune responses and enhance the efficacy of chemotherapy, thereby potentially improving the therapeutic outcomes of colorectal cancer [9–11].

Amidst the complex interplay between microbiota and cancer, a paradigm shift has occurred, heralding the advent of antitumor therapies that manipulate tumor-associated bacteria. These include prebiotics, probiotics, antibiotics, phage navigation technologies, and fecal microbiota transplantation [12–15]. Studies have shown that probiotic nanoparticles (NPs) loaded with capecitabine improved the treatment of colorectal cancer by combining intestinal microbiota modulation with chemotherapy [16]. Additionally, magnetic natural lipid NPs have been used for oral treatment of colorectal cancer, potentiating antitumor immunity and regulating microbiota metabolites [17]. Inhalable capsular polysaccharide-camouflaged gallium-polyphenol NPs have been shown to enhance lung cancer chemotherapy by depleting local lung microbiota [18]. Furthermore, fecal microbiota transplantation has been found to improve the efficacy of tirilizumab and furaquintinib in colorectal cancer by remodeling the microbial environment [19]. Combination therapy with the antibiotic metronidazole has been shown to inhibit the growth of colorectal cancer enriched with *F. nucleatum* in a xenograft mouse model [20]. Notably, the use of antibiotic silver-tinidazole complex encapsulated in liposomes effectively eliminated the tumor-promoting bacteria *F. nucleatum* in primary tumors and liver metastases, leading to the inhibition of colorectal tumor growth and metastasis [21]. Moreover, phage-navigated NPs have been used for targeted delivery of chemotherapeutic drug irinotecan to colorectal tumors and eliminate intratumoral *F. nucleatum* to enhance the chemotherapeutic effect [22]. Inactivated *Peptostreptococcus anaerobius*, which can be specifically enriched into colorectal tumors, have been used as bionic carriers to target the tumors enriched with bacterial microbiota, thereby exerting a synergistic antimicrobial and chemotherapeutical effect [23].

Oral colon-targeted drug delivery system is considered as a promising approach for directly targeting gastrointestinal diseases. However, a series of physical, chemical, and enzymatic barriers hinder the delivery and stability of orally administered drugs within the gastrointestinal tract. Gastric acid and numerous enzymes in the gastrointestinal tract, including glucosidase, pepsin, and trypsin, are the primary obstacles to colon-specific delivery [24,25]. Inulin, as a formulation ingredient approved by Food and Drug Administration for both intravenous and oral drug delivery [26–28], can protect drugs from the acidic and enzymatic environment in the upper gastrointestinal tract, ensuring their degradation by beneficial bacteria in the colon for colon-specific drug release [29–31]. Moreover, inulin-based orally administrated gels exhibit bioadhesive properties, prolonging gastric and intestinal emptying through thickening effects and adhering to the intestinal mucosal layer, thereby increasing retention and accumulation in the colon [32]. On the other hand, accumulating evidences indicate that inulin undergoes targeted degradation by advantageous bacteria in the colon, yielding substantial SCFAs. These SCFAs exert probiotic-like effects by shaping the intestinal microenvironment: suppressing harmful bacteria, bolstering beneficial bacteria, and facilitating microbiota restoration [22,33–37]. Moreover, SCFAs are key messengers between the intestinal microbiota and the host immune system [38], capable of triggering a series of antitumor immune responses by binding to G protein-coupled receptor 43 (GPR43) in the intestines [32] and activating the histone deacetylases/ID2/interleukin-12 (IL-12) receptor pathway in CD8^+^ T cells [39].

**Fig. 1.**
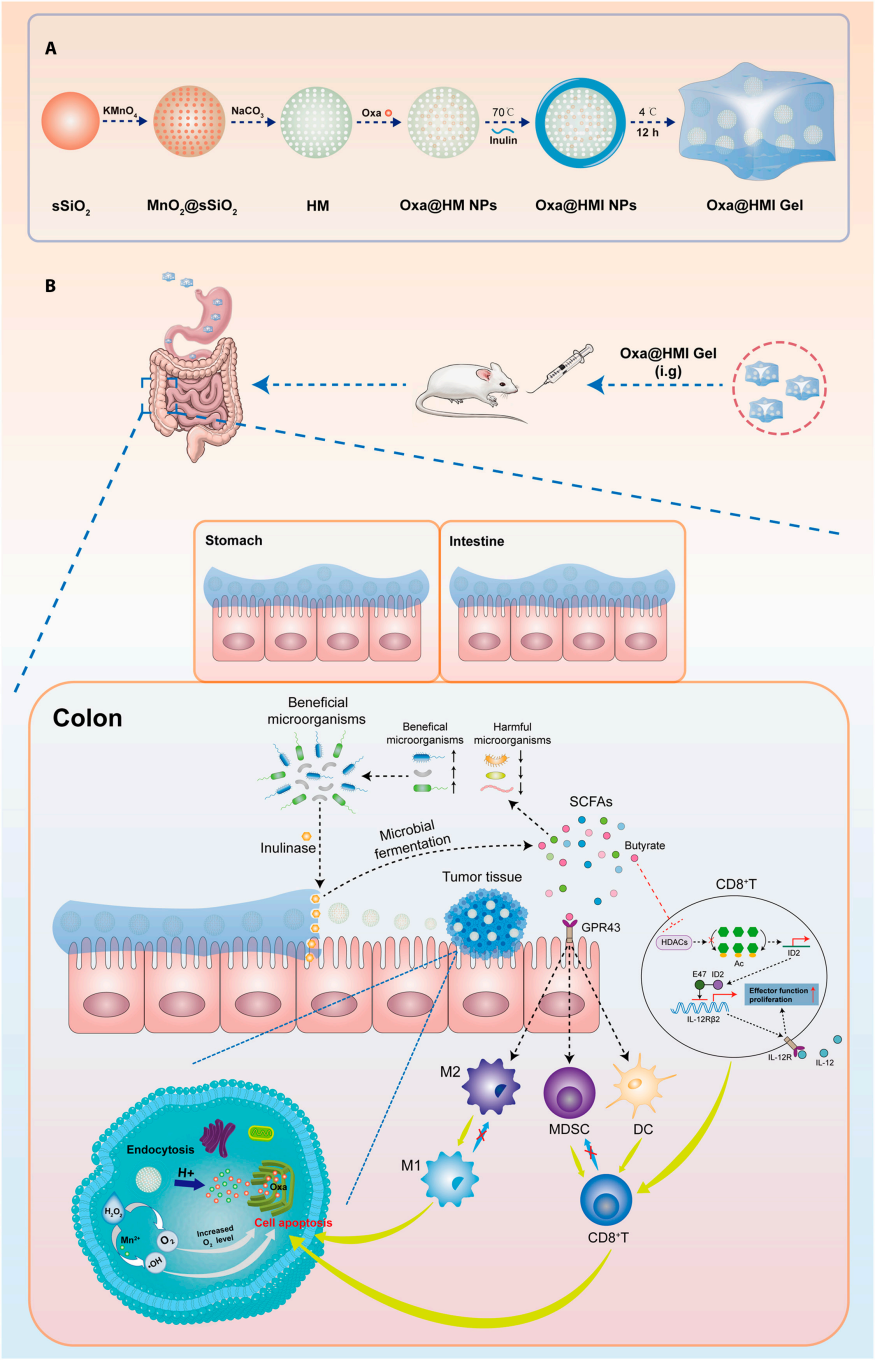
(A) A schematic diagram indicating the step-by-step synthesis of Oxa@HMI Gel. (B) Colon tumor suppression mechanism of Oxa@HMI Gel via regulating microbiota and immune microenvironment.

In this study, we developed an orally administrated hydrogel containing hollow MnO_2_ (HM) NPs loaded with the chemotherapeutic drug Oxa (Oxa@HMI) (Fig. 1A). The inulin gelatinous matrix promoted the accumulation and concentration of the active ingredient at the lesion site through enhancing bioadhesion and prolonging colon retention. This facilitated the specific degradation of inulin in Oxa@HMI by inulinase secreted by beneficial bacteria in the colon, leading to the production of SCFAs. The SCFAs, in turn, initiated a cascade of antitumor immune responses by modulating the balance between harmful and beneficial bacteria. On the other hand, degradation of the inulin gel matrix facilitated the colorectal cancer site-targeted exposure of internal nanomedicine Oxa@HM, which was degraded in the acidic tumor microenvironment (TME) to generate Mn^2+^ and O_2_ and release the loaded Oxa. The generated Mn^2+^ and O_2_ further exacerbated the oxidative stress levels in the TME through generating reactive oxygen species (ROS) to achieve effective tumor growth inhibition (Fig. 1B). The Oxa@HMI hydrogel with bacteria-targeted drug release can promote chemotherapy efficiency and activate antitumor immune responses by interfering with the microbial environment within the tumor tissues. This approach offers a promising therapeutic strategy for microbial-invasive colorectal cancer.

## Results

### Preparation and characterization of Oxa@HMI NPs

**Fig. 2.**
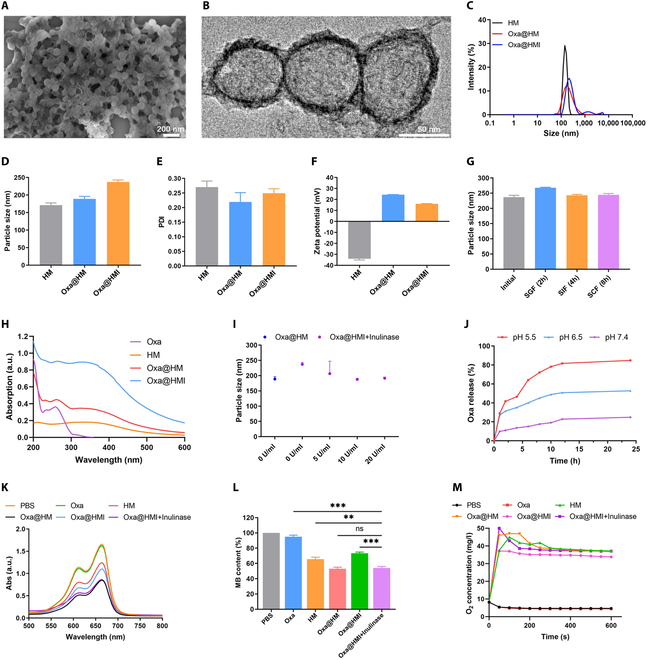
Preparation and characterization of Oxa@HMI NPs. (A) SEM image of Oxa@HMI NPs. (B) TEM image of Oxa@HMI NPs. (C) Size distribution of various NPs evaluated by dynamic light scattering analysis. Particle size (D), PDI (E), and zeta potential (F) of HM NPs, Oxa@HM NPs, Oxa@HMI NPs, respectively (*n* = 3). Particle size (G) of Oxa@HMI NPs after exposure to SGF, SIF, and SCF for 2, 4, and 8 h, respectively (*n* = 3). (H) UV-vis spectra of different solutions. (I) Particle size of NPs with or without inulin coating at conditions with inulinase of different concentrations (*n* = 3). (J) TME-responsive Oxa release profiles of Oxa@HMI NPs (with inulinase) at different pH values. UV-vis spectra (K) and representative absorption (L) of MB that degraded due to the •OH generated from various solutions with or without presence of inulinase. (M) O_2_ generation ability of different treatments in presence of H_2_O_2_ (100 μM). The data are presented as mean ± SD. **P < 0.01, ***P < 0.001; ns, no significance.

The synthesized Oxa@HMI NPs appeared spherical morphology according to scanning electron microscopy (SEM) observation (Fig. 2A); meanwhile, their hollow structure and uniform surface coating with inulin were clearly seen though transmission electron microscopy (TEM) images (Fig. 2B). The dynamic light scattering detection showed the nanoscale and well->dispersed particles in various solutions with particle sizes ranged from 170 to 240 nm (Fig. 2C to E). Obviously, after loading Oxa (Oxa@HM) or modifying inulin on the surface (Oxa@HMI), there were corresponding increases on particle sizes, also demonstrating the successful construction of the core-layered structure. Meanwhile, the zeta potential of HM NPs has converted from negative to positive during the loading and coating process, which also confirmed the successful synthesis of Oxa@HMI NPs (Fig. 2F). In addition, with the alkaline environment of the intestinal tract, the mucin layer comprising the intestinal mucus is characterized by a negative charge [40], thereby enabling electrostatic interactions with positively charged Oxa@HM NPs and Oxa@HMI NPs, facilitating their enteral absorption. According to the ultraviolet–visible (UV-vis) spectrum (Fig. 2H), characteristic absorption peaks of Oxa were observed in Oxa@HM NPs and Oxa@HMI NPs, indicating the efficient loading of Oxa onto these NPs.

### Gastrointestinal stability and TME-responsive disintegration of Oxa@HMI NPs

Given the necessity to maintain optimal physicochemical stability in the gastrointestinal milieu, it was imperative for Oxa@HMI NPs to enable efficient transportation to colonic lesion while preserving their structural integrity. Subsequently, the outer layer composed of inulin would undergo degradation by inulinase, an enzyme secreted by beneficial intestinal microbiota within the TME [41]. This enzymatic degradation would lead to the exposure of the HM core, inducing carrier disintegration and triggering the release of Mn^2+^, O_2_, as well as the encapsulated Oxa. Consequently, a comprehensive exploration of the stability of Oxa@HMI NPs within the gastrointestinal tract and the microbiota-abundant environment holds utmost meaning in understanding the mechanism of this therapeutic system in vivo.

We firstly assessed the physical stability of Oxa@HMI NPs in solutions mimic different gastrointestinal conditions (Fig. 2G and Fig. S1A and B). The particle size and polydispersity index (PDI) of Oxa@HMI NPs showed no significantly changes in simulated gastric fluid (SGF), simulated intestinal fluid (SIF), and simulated colonic fluid (SCF), respectively, confirming the good physical stability of Oxa@HMI NPs in gastrointestinal system during oral administration. However, the surface zeta potential of Oxa@HMI NPs significantly fluctuated after coincubation with SGF and SIF, possibly due to the influence of environmental factors such as pH value, ions, and salt effect. Subsequently, we simulated the microbiota-rich environment in colorectal cancer by adding inulinase. The particle size of Oxa@HMI NPs was gradually decreased with increasing inulinase concentration in the environment until it matched that of NPs without inulin coating (Oxa@HM) (Fig. 2I). This size decrease was attributed to the degradation of the outer inulin layer, leading to the complete exposure of the internal HM NPs. These findings demonstrated that Oxa@HMI NPs can maintain structural stability in simulated gastrointestinal solutions, allowing the delivery of intact NPs to colorectal tumor sites. Following that, the outer inulin protective layer would be degraded in a simulated tumor tissue microbiota environment, enabling the full exposure of the internal HM NPs and facilitating the TME-responsive disintegration and release of O_2_ and Oxa within the tumor tissue.

The drug release profiles of Oxa@HMI NPs (coincubated with inulinase) were recorded under conditions simulated various physiological environments in presence of inulinase (Fig. 2J). Compared with release behavior of Oxa at normal physiological condition (pH 7.4), the release rate in acidic solutions simulated TME (pH 6.5) was significantly higher, and this release was further accelerated in condition simulated lysosomal environment (pH 5.5). This observation indicated that the HM NPs exposed after the degradation of the inulin layer possessed excellent acid-responsive degradation properties, whereby HM was decomposed into Mn^2+^ and O_2_, as well as released the loaded Oxa. The released Mn^2+^ along with Oxa further produced ROS and O_2_ through the Fenton reaction, thereby upregulating the lipid peroxides level and causing damage to biomolecules such as lipid, protein, and DNA [42,43]. The ability of generated Mn^2+^ to mediate ROS production was evaluated using the methylene blue method. As shown in Fig. 2K and L, the phosphate-buffered saline (PBS) and Oxa groups exhibited minimal ROS production, while the HM NPs demonstrated a certain level of ROS generation. Remarkably, the Oxa@HM NPs exhibited even higher ROS yield, indicating the synergistic pro-oxidative effect of enzymatic-like activity by Oxa [44–46]. It is worth noting that the modification of inulin layer protected Oxa@HMI from erosion by acidic environments such as gastric fluid, thereby also limiting the ROS generation capability. This protective effect and limitation were reversed in the presence of inulinase (Oxa@HMI+Inulinase group). As previously mentioned, the process of the carrier degrading and Fenton reaction were accompanied by the production of O_2_. As shown in Fig. 2M, the PBS and Oxa groups hardly produced O_2_, while the HM-based groups produced up to 45 mg/l of O_2_ within less than 100 s. Similarly, the efficiency of O_2_ production was slightly slowed down in Oxa@HMI NPs group due to inulin protection, which was reversed in inulinase-present conditions.

### Intracellular biofunctions of Oxa@HMI NPs

**Fig. 3.**
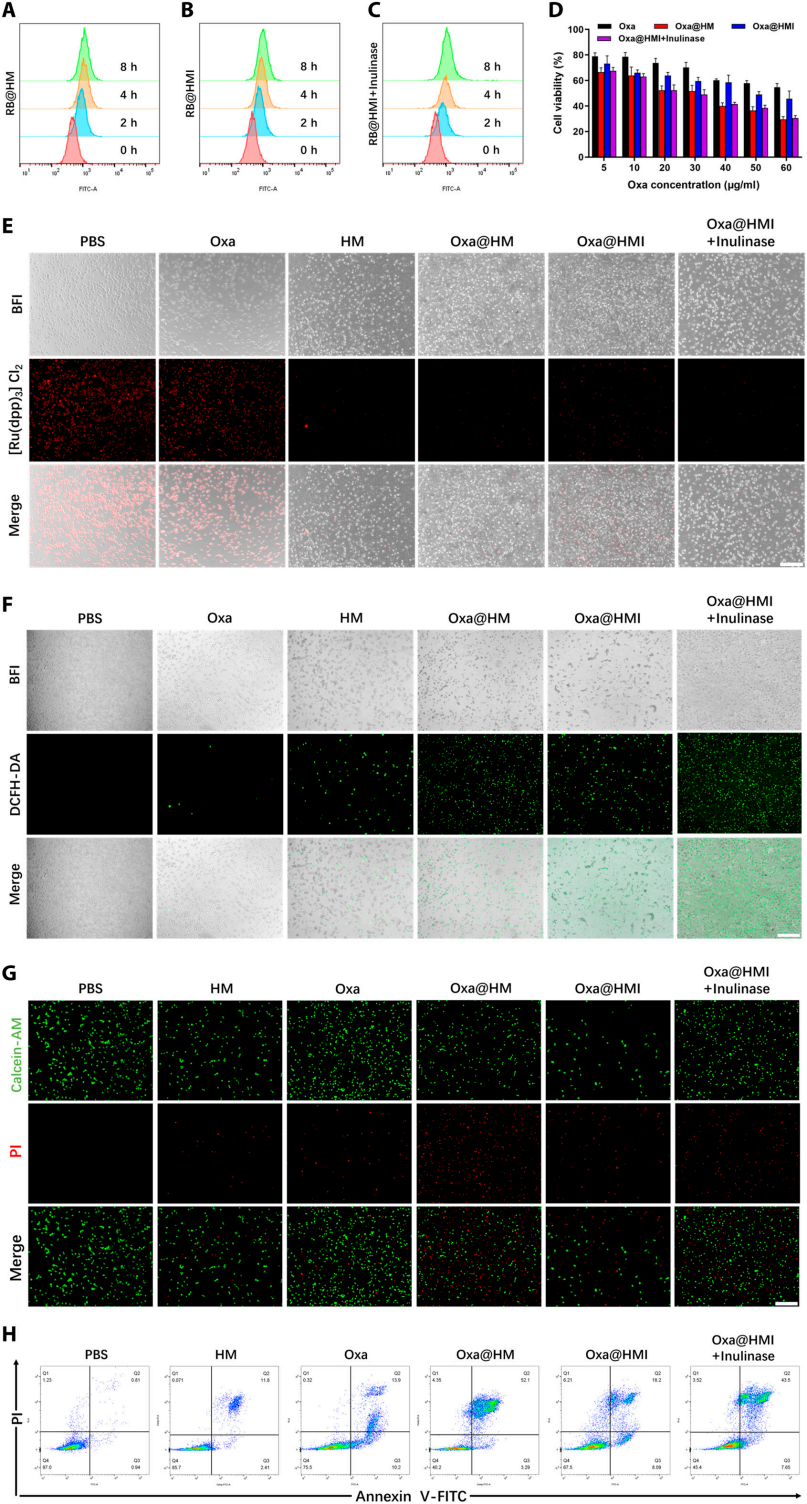
In vitro biofunctional evaluation of Oxa@HMI NPs on CT26 cells. Cellular uptake of RB@HM NPs (A), RB@HMI NPs (B), and RB@HMI NPs with inulinase (C) in CT26 cells analyzed by flow cytometry after incubation for 0, 2, 4, and 8 h, respectively (RB, Rhodamine B). (D) Cell viabilities of CT26 cells after 24 h incubated with various solutions at different concentrations (*n* = 5). (E) Representative CLSM images of intracellular O_2_ generation in CT26 cells after incubation with different solutions for 12 h under the hypoxic conditions. [Ru(dpp)_3_]Cl_2_ was used to indicate hypoxic levels. Scale bar: 10 μm. (F) Representative CLSM images of intracellular ROS generation in CT26 cells after incubation with different solutions for 24 h. DCFH-DA was used as ROS probe. Scale bar: 10 μm. (G) The CT26 cell viabilities evaluated by Live/Dead staining assay. Calcein AM: live cells. PI: dead cells. Scale bar: 10 μm. (H) Apoptosis of CT26 cell after different treatments analyzed by flow cytometry assay. The data are presented as mean ± SD.

In order to investigate the cellular uptake of Oxa@HMI NPs by CT26 cells, we utilized fluorescent Rhodamine B (RB)-modified NPs and analyzed the uptake of by CT26 cells at various incubation time points using flow cytometry (Fig. 3A to C). The intracellular accumulation of the NPs in CT26 cells was increased over time. After 8 h of incubation, the uptake of RB@HM NPs by CT26 cells was significantly higher compared to free RB, indicating that the nanocarriers markedly enhanced the efficiency of drug internalization into CT26 cells (Fig. S2). However, the uptake of RB@HMI NPs was slightly lower than that of RB@HM NPs, possibly due to the encapsulation of inulin on the outer layer of RB@HMI NPs hindering cellular uptake. Once the inulin layer was degraded, the cellular uptake of Oxa@HMI NPs became comparable to that of RB@HM NPs. This suggested that the protective outer inulin layer effectively prevented nontargeted internalization of NPs at microbiota-poor regions.

The cytotoxicity of the internalized NPs on CT26 cells was evaluated using MTT assay. The NP-based group exhibited significantly higher cytotoxicity than free Oxa, benefiting from the enhanced intracellular drug accumulation (Fig. 3D). Additionally, after internalization into tumor cells, HM nanocarriers were further degraded into Mn^2+^ and catalyzed the generation of ROS, synergistically enhancing the oxidative stress levels within tumor cells in coordination with Oxa. However, the use of blank carriers HM NPs alone exhibited good biocompatibility and did not cause oxidative damage to CT26 cells (Fig. S3). The limited cytotoxicity of Oxa@HMI NPs on tumor cells can be attributed to the hindered cellular uptake caused by the outer coating of inulin, and the protection effect on HM NPs core from degradation in acidic environments, which was consistent with the cellular uptake results.

To further understand the mechanisms underlying the cytotoxicity induced by various NP groups, we assessed the O_2_ level generated by Oxa@HMI NPs in response to acidic lysosomal environment using the hypoxia indicator [Ru(dpp)_3_]Cl_2_ [47]. Under hypoxic conditions, CT26 cells treated with PBS and free Oxa exhibited strong red fluorescence indicating hypoxia, while CT26 cells treated with HM-containing NPs showed much weaker fluorescence signal, indicating that HM nanocarriers were successfully disassembled and produced O_2_ within cells (Fig. 3E). The production of ROS in CT26 cells treated with different NP formulations was consistent with the O_2_ generation capacity (Fig. 3F). HM-containing nanocarriers demonstrated effective ROS generation, with Oxa@HM NPs and Oxa@HMI NPs+Inulinase groups showing more pronounced effects, attributed to the enzymatic activity of Oxa and the synergistic catalytic effect MnO_2_ in ROS production. The results of live/dead cell staining (Fig. 3G) and flow cytometry apoptosis assay (Fig. 3H) showed that HM-containing NPs, which possessed efficient cellular uptake and the ability to generate O_2_ and ROS within cells, exhibited higher apoptosis and lower cell viability, consistent with the results of MTT cell viability assay.

### Construction of Oxa@HMI hydrogel and its biodistribution in orthotopic colorectal >tumor-bearing mice

**Fig. 4.**
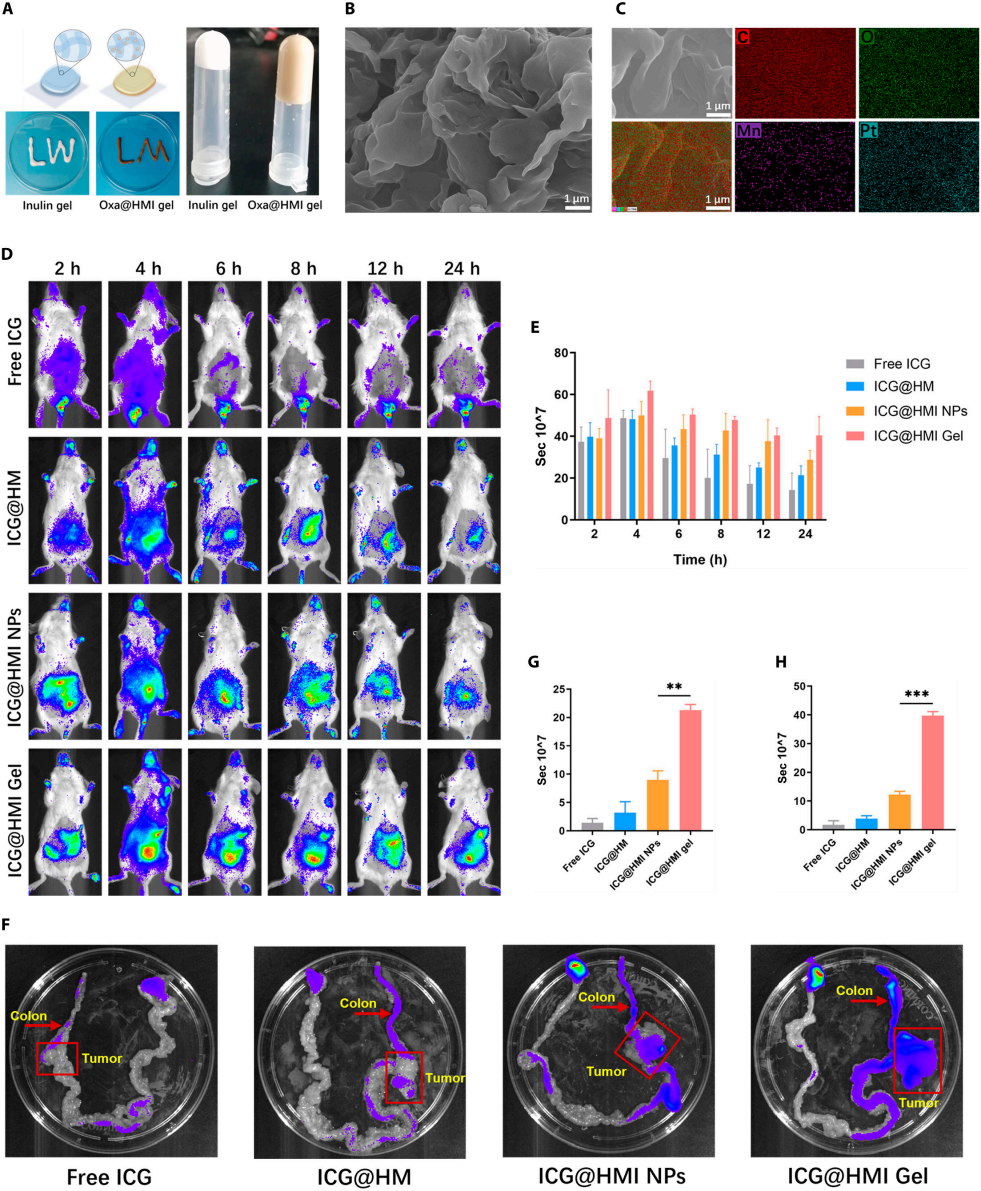
Construction of Oxa@HMI Gel and its biodistribution in orthotopic colorectal tumor-bearing Balb/c mice. (A) Schematic and digital photos of inulin gel before and after crosslinking with NPs. SEM image (B) and the corresponding element mappings (C, O, Mn, and Pt) (C) of Oxa@HMI Gel. Representative in vivo fluorescence images of tumor-bearing mice (D) and the corresponding quantitative estimation (E) at different time intervals (2, 4, 6, 8, 12, and 24 h) after oral administration with free ICG, ICG@HM NPs, ICG@HMI NPs, and ICG@HMI Gel (*n* = 3). (F) Ex vivo fluorescence images of gastrointestinal tract collected for mice at 24 h after oral administration with various formulations. The corresponding quantification of the fluorescence intensity in colons (G) and colon tumors (H) tissues (*n* = 3). The data are presented as mean ± SD. **P < 0.01, ***P < 0.001.

To facilitate oral administration and gastrointestinal drug delivery, Oxa@HMI hydrogel was constructed by adjusting the proportion of inulin modification on Oxa@HMI NPs to achieve better adhesion onto the intestinal mucosal layer and prolonged intestinal retention time. The viscous Oxa@HMI hydrogel exhibited low fluidity when converted placed or placed on the flat plate (Fig. 4A). SEM images confirmed the porous and loose structure of the Oxa@HMI hydrogel (Fig. 4B). Meanwhile, elemental mapping data revealed the uniform distribution of Pt and Mn from Oxa and HM nanocarrier, respectively (Fig. 4C). The effective encapsulation efficiency (64.5 wt%) and the drug loading capacity (45.0 wt%) of Oxa in Oxa@HMI hydrogel were determined through inductively coupled plasma-mass spectrometry (ICP-MS).

We established an orthotopic colorectal tumor model by injecting CT26-Luc cells into the submucosa of ascending colon wall of mice. Fluorescent probe indocyanine green (ICG) was used to label different materials to evaluate their distribution and retention after oral administration in orthotopic colorectal tumor-bearing mice. As shown in Fig. 4D and E, the ICG fluorescence from ICG@HM accumulated in the intestinal tract of mice within a short time; however, it was rapidly degraded and cleared due to the lack of inulin shell protection in the gastrointestinal environment, resulting in no effective tumor accumulation observed at 24 h, which occasion was similar to free ICG. In contrast, ICG@HMI NPs and ICG@HMI hydrogel, which were protected by an inulin layer on the surface, showed significantly longer intestinal retention time. Importantly, fluorescence imaging of excised tissues at 24 h revealed that both ICG@HMI NPs and hydrogel maintained relatively high drug accumulation in the intestinal tumor tissue compared to other parts of intestine. The hydrogel group exhibited even higher drug amount, 3 times that of the NP group (Fig. 4F to H).

Surface modification of inulin served as a protective layer to shield acid-sensitive degradable HM NPs from the acidic environment of the gastrointestinal tract during oral administration. Once the materials reached the colorectal region, they are selectively degraded by inulinase produced by beneficial bacteria, thereby exposing the internal nanomedicine. This approach effectively enhanced the stability of the drug carrier in the gastrointestinal system and promoted specific release in colorectal tumors. Furthermore, by adjusting the proportion of inulin modification to form the ICG@HMI hydrogel, it would gain prolonged gastric emptying by thickening effect and adhesion to the intestinal mucosal layer due to the polysaccharide fibers in hydrogel matrix, further enhanced the accumulation and retention in colorectal tumors [48]. Compared to using inulin alone, the ICG@HMI hydrogel based on NPs inside exhibited a longer retention time in the colon (and cecum) in a gel strength-dependent manner, which further increased the cumulative exposure of inulin in the colon [32].

### Antitumor efficacy of Oxa@HMI hydrogel on orthotopic colorectal tumor-bearing mice

**Fig. 5.**
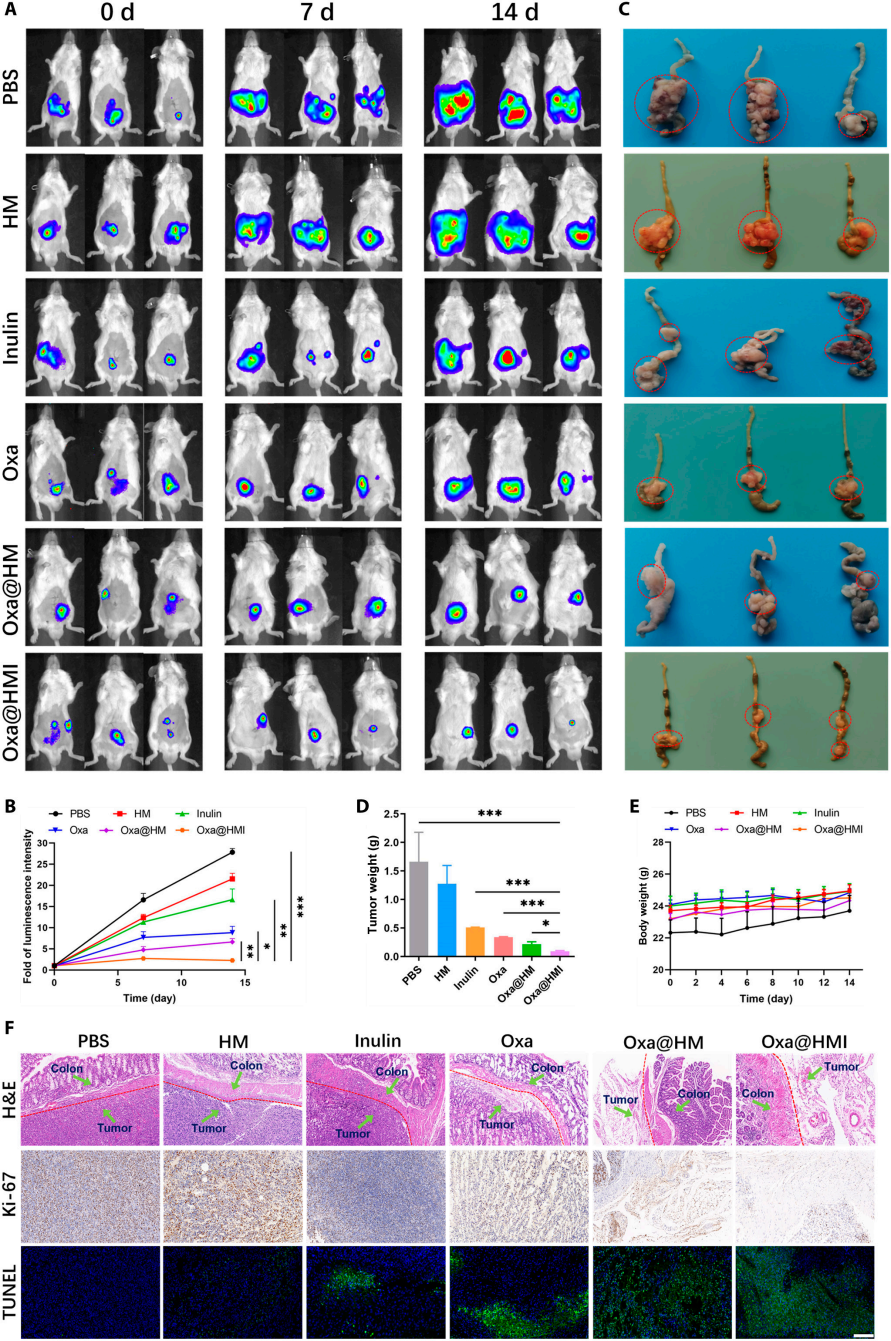
In vivo antitumor activity of Oxa/HMI Gel on orthotopic colorectal tumor-bearing Balb/c mice. In vivo bioluminescence images (A) and corresponding quantification (B) of orthotopic colorectal tumor-bearing mice receiving various treatments (PBS, HM NPs, Inulin Gel, Oxa@HM NPs, and Oxa@HMI Gel) at 0, 7, and 14 d (*n* = 3). (C) Representative images of colon tumors excised from the sacrificed tumor-bearing mice. Red circles indicated for colorectal tumor regions. Tumor weight (D) and body weight (E) of orthotopic colorectal tumor-bearing mice after various treatments (*n* = 3). (F) Histological analysis by H&E, Ki-67, and TUNEL staining of tumor sections after various treatments. Scale bar: 20 μm. The data are presented as mean ± SD. *P < 0.05, **P < 0.01, ***P < 0.001.

To investigate the therapeutic efficacy of Oxa@HMI hydrogel in tumors, an orthotopic colorectal tumor model was established by injecting CT26-Luc cells into the colonic mucosa of mice. The tumor-bearing mice were orally administered various formulations for treatment, and the tumor volume and size were dynamically monitored using in vivo imaging system (IVIS). As shown in Fig. 5A and B, compared to the PBS group, HM NPs exhibited only a slight antitumor effect due to its vulnerability to degradation in acidic gastrointestinal environment and limited in vivo retention. The Inulin gel group demonstrated a moderate antitumor activity, potentially attributed to the fermentation and degradation of inulin gel by colonic beneficial bacteria, leading to the production of SCFAs and triggering antitumor immune responses in the host. Notably, the Oxa@HMI hydrogel group exhibited significantly greater inhibition of tumor growth, as evidenced by visibly smaller tumor size and weight compared to the clinical first-class chemotherapeutic drug Oxa and the Oxa-loaded NPs Oxa@HM (Fig. 5C and D). This superior outcome can be attributed to the specific exposure of the internal HM core to the tumor tissue, subsequent degradation in the acidic TME, and release of Oxa, Mn^2+^, and O_2_ into the surrounding circumstance. The synergistic effect of these factors enhanced tumor oxidative stress levels and improved chemotherapy sensitivity. Furthermore, histological hematoxylin and eosin (H&E) staining of tumor tissues treated with different strategies revealed the lowest tumor cell density in the Oxa@HMI hydrogel group (Fig. 5F). Immunohistochemical analysis demonstrated reduced proliferation (Ki-67 positive) and increased apoptosis (terminal deoxynucleotidyl transferase dUTP nick end labeling [TUNEL] positive) signals in the tumor cells of the Oxa@HMI hydrogel treatment group, further substantiating the efficacy of this treatment approach in promoting tumor cell apoptosis and inhibiting tumor growth.

### Oxa@HMI hydrogel normalized the intestinal microbiota in orthotopic colorectal >tumor-bearing mice

Numerous studies have confirmed that the composition of the microbiota in the intestine and tumor influence the development and treatment of colorectal cancer [33,49]. Probiotics have been found to exert crucial effects in modulating the gut microbiome. They suppress the proliferation of pathogenic bacteria, foster the expansion of beneficial bacteria, and promote the restoration of intestinal microbial homeostasis. These effects are attributed to the secretion or fermentation of substrates by probiotics, resulting in the generation of SCFAs [22,33–37]. The inulin composition in Oxa@HMI hydrogel is one of the main carbohydrates that act as the prebiotics [32,50]. To explore the role of inulin in tumor suppression and microbiota normalization within the therapeutic system, fecal samples and tumor tissues were collected from each group of tumor-bearing mice after the treatment process. High-throughput 16S ribosomal ribonucleic acid gene sequencing was conducted to evaluate the variation of microbiota in the intestinal tract and tumor tissues respectively, aiming to explore the potential mechanisms by which Oxa@HMI hydrogel exerted antitumor effects through modulation of microbial composition.

**Fig. 6.**
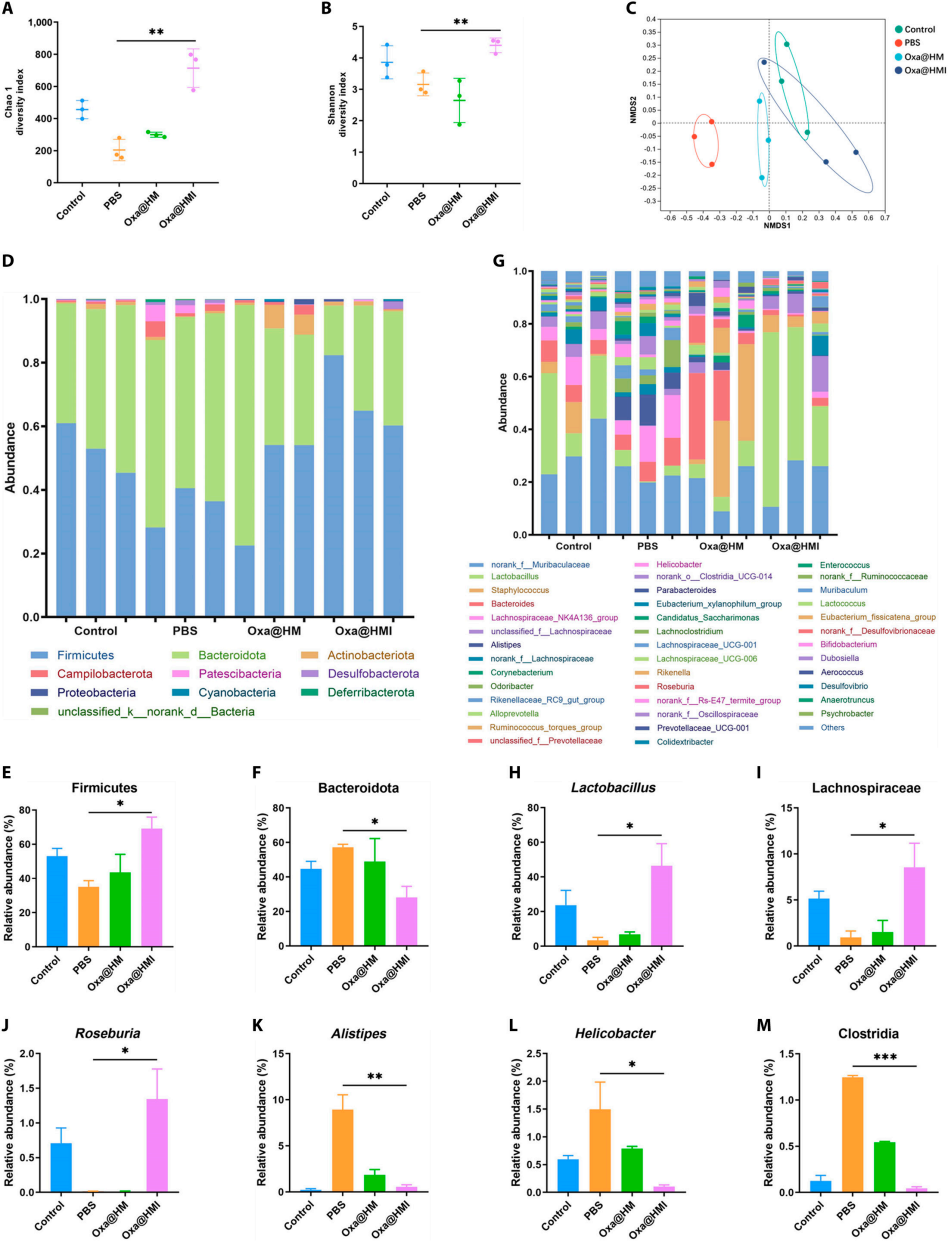
The gut microbiota regulation by Oxa@HMI Gel on orthotopic colorectal tumor-bearing Balb/c mice. The richness and diversity analysis of gut microbiota from various treated mice expressed as Chao 1 (A) and Shannon (B) indices. (C) NMDS analysis of the gut microbial composition. Composition of the gut microbiota at phylum level (D) and genus level (G), respectively (*n* = 3). Relative abundance of Firmicutes (E) and Bacteroidota (F) at the phylum level (*n* = 3). Relative abundance of beneficial gut microbiota including *Lactobacillus* (H), Lachnospiraceae (I), and *Roseburia* (J) at the genus level (*n* = 3). Relative abundance of harmful gut microbiota including *Alistipes* (K), *Helicobacter* (L), and Clostridia (M) at the genus level (*n* = 3). The data are presented as mean ± SD. *P < 0.05, **P < 0.01, ***P < 0.001.

The results of fecal analysis in orthotopic colorectal tumor-bearing mice were shown in Fig. 6A and B and Fig. S4. The α-diversity indices, including the Shannon index, Chao-1 index, and Sobs index, reflect the diversity and richness of the microbiota, respectively [51]. The Chao-1, Shannon, and Sobs indices in the PBS group were significantly decreased, indicating a substantial alteration in the intestinal microbiota of the lesion site, characterized by reduced richness and diversity. In the group of mice orally administered Oxa@HMI hydrogel, the richness and diversity of the intestinal microbiota were significantly increased; however, this phenomenon was not observed in the Oxa@HM group without inulin modification. Subsequently, nonmetric multidimensional scaling (NMDS) analysis was conducted using weighted UniFrac distances to visualize the similarity of intestinal microbial composition among the tested groups. Compared to the healthy mice (Control group), the composition of the intestinal microbiota in the mice affected by orthotopic tumors (PBS group) underwent the most significant changes. However, oral administration of Oxa@HMI hydrogel transformed the microbial composition from the dysbiosis observed in the PBS group mice to a homeostasis that of the healthy mice (Fig. 6C). This transformation can be attributed to the fermentation of inulin fibers in the Oxa@HMI hydrogel by intestinal bacteria, resulting in the production of a large amount of SCFAs. This selective regulation of beneficial and harmful bacteria in the disrupted microbial community increased the richness and diversity of microbiota [52]. Furthermore, this prebiotic effect promoted the restoration of the intestinal microbiota and achieved microbiota normalization [32,50].

We further analyzed the composition of intestinal microbiota at phylum/family/genus levels in each group. Compared to PBS and Oxa@HM NPs groups without inulin, oral administration of Oxa@HMI hydrogel significantly increased the relative abundance of beneficial Firmicutes phylum (Fig. 6D and E), including probiotics represented by genera *Lactobacillus*, Lachnospiraceae family, and *Roseburia* genus (Fig. 6G to J). On the other hand, the relative abundance of pathogenic bacteria representative of the Bacteroidota phylum was significantly reduced (Fig. 6D and F).

Firmicutes and Bacteroidota are 2 dominant bacteria in the intestinal microbiota, and their balance may play a crucial role in host physiology [53]. Among them, Firmicutes and its representative genera such as *Lactobacillus*, Lachnospiraceae, and *Roseburia* are widely recognized as beneficial bacteria in the intestine. They play important roles in inhibiting tumor progression and promoting antitumor efficacy by producing SCFAs such as acetate, propionate, and butyrate [54–56]. SCFAs have been evidenced to enhance the effectiveness of immunotherapy and Oxa-based chemotherapy [32,39]. The decreased relative abundance of SCFAs-producing bacteria is considered as a typical characteristic of intestinal microecological dysbiosis in mice with colitis and colitis-associated colorectal cancer [57]. Moreover, these bacterial genera are associated with clinical responses in patients receiving ICB therapy [58–61]. Researchers have demonstrated that altering the composition of the mice intestinal microbiota, through a high-fiber diet, such as inulin supplementation, promoted SCFAs production and inhibited colorectal cancer occurrence [62,63].

On the other hand, we observed that following oral administration of Oxa@HMI hydrogel, the relative abundance of harmful bacteria associated with tumor progression in the intestines of orthotopic colorectal tumor-bearing mice, such as *Alistipes*, *Helicobacter*, and Clostridia, significantly decreased, even reaching levels comparable to those of healthy mice (Fig. 6G and K to M). Extensive literatures have demonstrated that *Alistipes* promotes the development of colorectal cancer by activating the IL-6/STAT3 signaling pathway [64]. *Helicobacter* is involved in chronic inflammation, promoting colorectal carcinogenesis by deregulating intestinal immunity and inducing degradation of the mucosal microbiota [65,66]. Clostridia induces amino acid degradation in colorectal cancer [67]. The results obtained above confirmed that inulin present in Oxa@HMI hydrogel, specifically degrades into SCFAs within the intestines, exerting a prebiotic effect by remarkably increasing the richness and diversity of beneficial bacteria while reducing harmful bacteria implicated in colorectal cancer development. This contributed to the normalization of the intestinal microbiota in orthotopic colorectal tumor-bearing mice and held therapeutic potential for colorectal cancer treatment.

### Oxa@HMI hydrogel reshaped the intratumoral microbiota in orthotopic colorectal >tumor-bearing mice

It has been reported that various types of bacteria have been found in most tumors and their adjacent normal tissues, primarily residing within cancer cells and immune cells, including colorectal cancer, pancreatic cancer, esophageal cancer, liver cancer, breast cancer, and lung cancer [68]. The intratumoral microbiota not only affects the efficacy of tumor chemotherapy and the host immune system but also influences the migration and colonization of tumor cells. Moreover, the intratumoral microbiota has now developed as a biomarker for the auxiliary diagnosis and treatment of tumors [69]. Therefore, we proceeded to investigate the impact of orally administrated Oxa@HMI hydrogel on the intratumoral microbiota in orthotopic colorectal cancer.

**Fig. 7.**
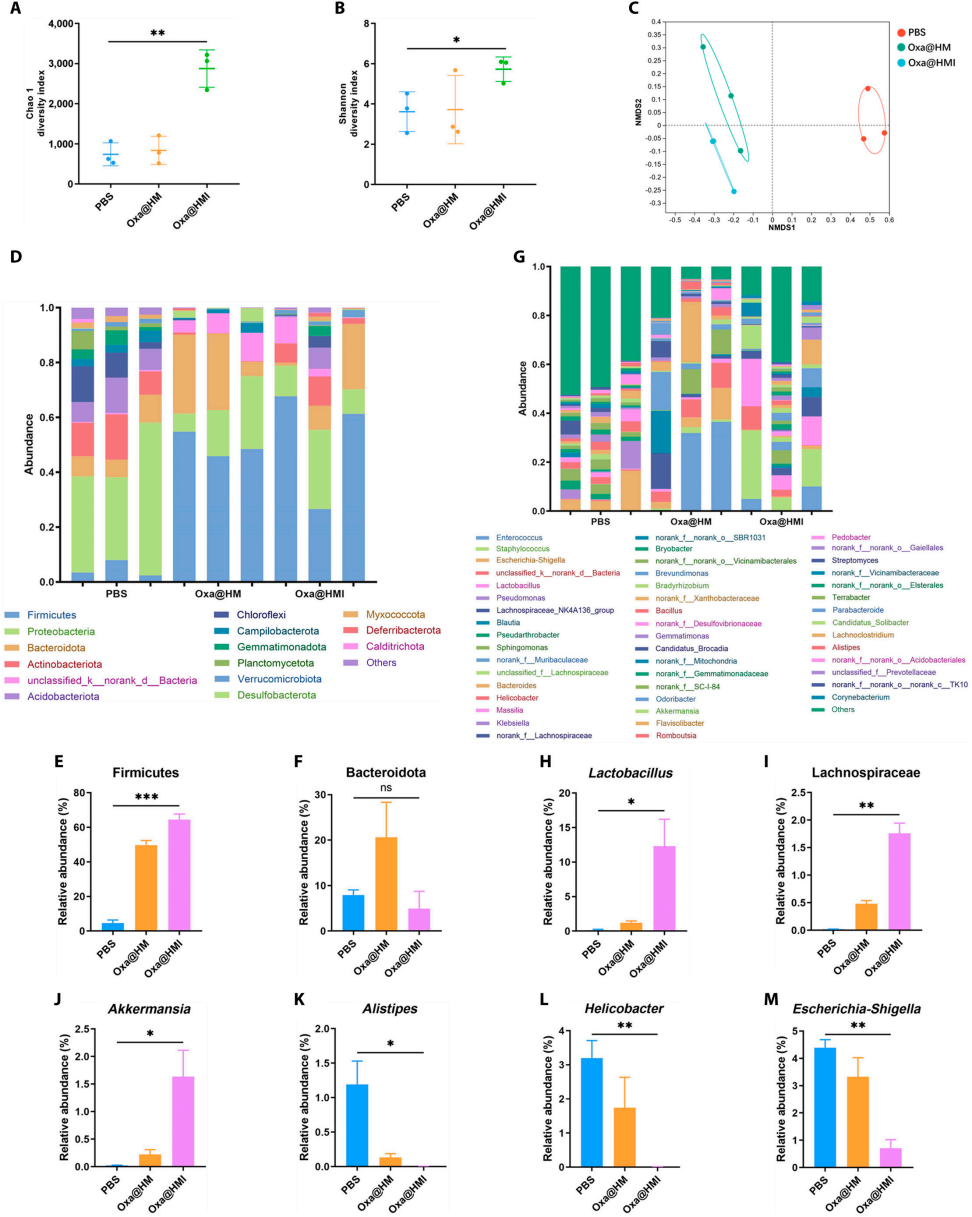
The intratumoral microbiota regulation by Oxa@HMI Gel on orthotopic colorectal tumor-bearing Balb/c mice. The richness and diversity analysis of intratumoral microbiota from various treated mice expressed as Chao 1 (A) and Shannon (B) indices. (C) NMDS analysis of the intratumoral microbial composition. Composition of the intratumoral microbiota at phylum level (D) and genus level (G), respectively (*n* = 3). Relative abundance of Firmicutes (E) and Bacteroidota (F) at the phylum level (*n* = 3). Relative abundance of beneficial intratumoral microbiota including *Lactobacillus* (H), Lachnospiraceae (I), and *Akkermansia* (J) at the genus level (*n* = 3). Relative abundance of harmful intratumoral microbiota including *Alistipes* (K), *Helicobacter* (L), and *Escherichia*-*Shigella* (M) at the genus level (*n* = 3). The data are presented as mean ± SD. *P < 0.05, **P < 0.01, ***P < 0.001; ns, no significance.

Consistent with the previous study on intestinal microbiota regulation, the Chao-1, Shannon, and Sobs indices in the tumor tissues of untreated mice (PBS group) were found to be extremely low, aligning with previous literature reporting the enrichment of functionally monotonous harmful bacteria within tumors. Following oral administration of Oxa@HMI hydrogel, these indices significantly increased, indicating an improved richness and diversity of the intratumoral microbiota (Fig. 7A and B and Fig. S5). NMDS analysis based on weighted UniFrac distances and microbial analysis at the phylum/family/genus levels revealed significant alterations in the microbial composition after Oxa@HMI hydrogel treatment compared to the pretreatment state (Fig. 7C). This difference was mainly concentrated in the significant increase in the abundance of beneficial, antitumor bacteria within the Firmicutes phylum, represented by the *Lactobacillus*, Lachnospiraceae, and *Akkermansia* (Fig. 7D, E, and H to J). Furthermore, we also observed a significant reduction in the relative abundance of harmful bacteria such as Bacteroidota, *Alistipes*, and *Helicobacter*, as well as suppression in the abundance of numerous pathogens originating from the *Escherichia* and *Shigella* genera within the tumor tissues after treatment (Fig. 7D, F, and K to M). These findings reconfirmed that the role of Oxa@HMI hydrogel in reshaping the microbiota, aiming to achieve colorectal cancer therapy by interfering microbial composition within intestines and solid tumors.

### Oxa@HMI hydrogel activated antitumor immune responses via normalizing microbiota

**Fig. 8.**
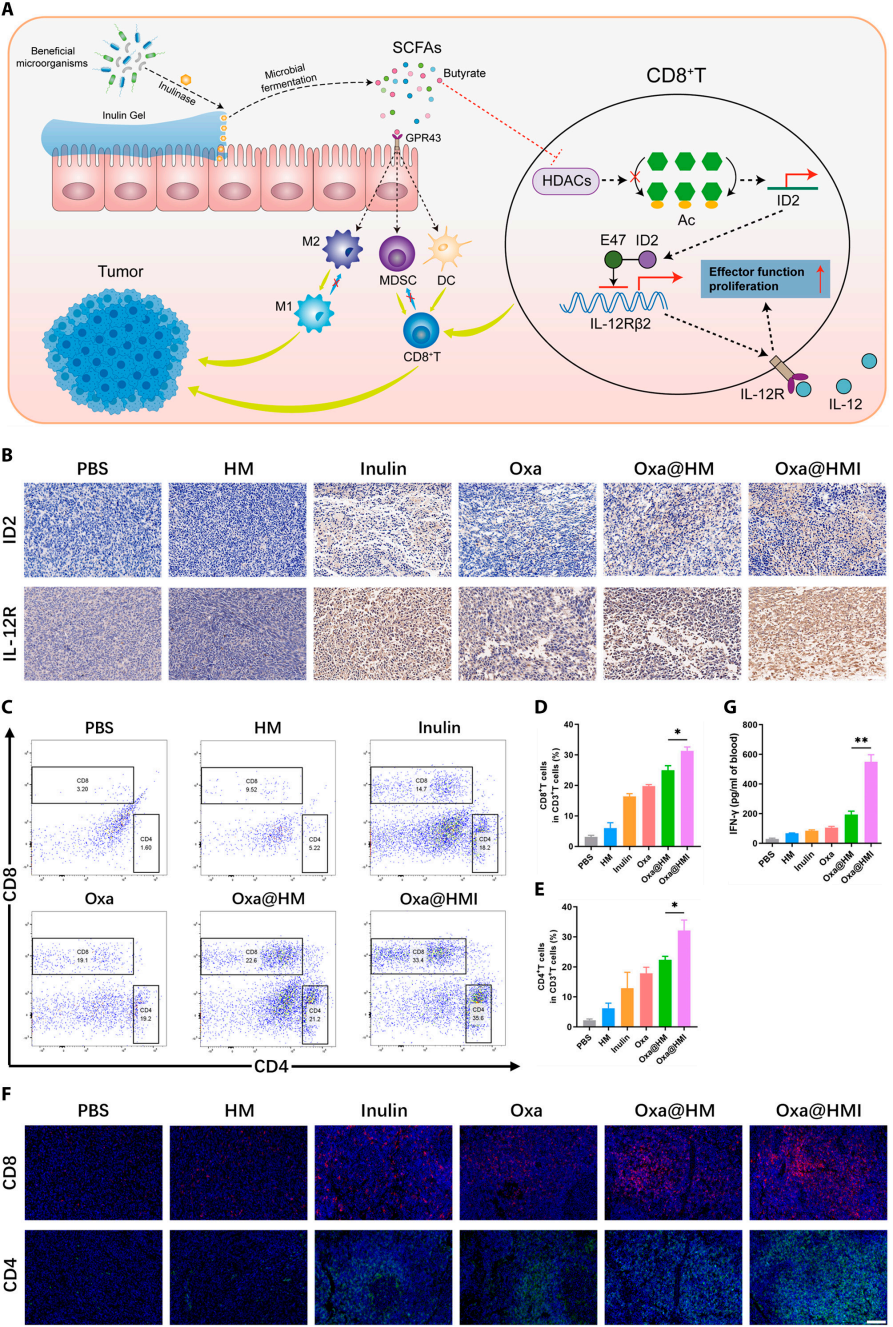
The tumor immune microenvironment modulation by Oxa@HMI Gel via regulating intratumoral microbiota on orthotopic colorectal tumor-bearing Balb/c mice. (A) Schematic diagram of tumor immune microenvironment modulation by Oxa@HMI Gel via regulating intratumoral microbiota. (B) Immunohistochemical analysis of ID2 and IL-12 receptors on T cells in TME of orthotopic colorectal tumor-bearing mice after various treatments. Scale bar: 50 μm. Representative flow cytometric images (C) and corresponding quantitative analysis of CD8^+^ T cells (D) and CD4^+^ T cells (E) infiltrated in the tumor tissues form mice after different treatments (*n* = 3). (F) Immunofluorescent staining images of CD8^+^ T cells and CD4^+^ T cells in tumor tissues from mice after various treatments. Scale bar: 20 μm. (G) The secretion levels of serum IFN-γ determined by enzyme-linked immunosorbent assay (*n* = 3). The data are presented as the mean ± SD. *P < 0.05, **P < 0.01.

Oxa@HMI hydrogel can selectively regulate beneficial and harmful bacteria to achieve microbiota normalization. Butyric acid, a metabolic byproduct by beneficial bacteria, can stimulate ID2 expression in CD8^+^ T cells via its inhibitory activity on histone deacetylases. Furthermore, the upregulated ID2 can induce IL-12 receptor expression on the surface of CD8^+^ T cells, thereby regulating IL-12 signaling to promote the infiltration of CD8^+^ T cells into tumor tissues and enhancing secretion of interferon-γ (IFN-γ), ultimately boosting T cell-mediated antitumor immune responses [39]. Additionally, SCFAs have been reported to bind to the GPR43 in the gastrointestinal tract, triggering a series of antitumor immune responses on colorectal tumor-bearing mice, markedly increasing the frequency of CD8^+^, IFN-γ^+^CD8^+^, and CD4^+^ T cells within the tumor tissues, activating dendritic cells (DCs) (CD86^+^CD11c^+^), promoting M1 polarization of macrophages, and reducing the frequency of myeloid-derived suppressor cells (MDSCs) (Fig. 8A) [32,70].

Therefore, we investigated the ability of Oxa@HMI hydrogel to regulate antitumor immune responses through microbiota normalization by examining the expression levels of corresponding receptors and immune cells in colorectal tumor-bearing mice. Tumor tissues from mice treated with inulin showed upregulation of ID2 and IL-12 receptors (Fig. 8B), in tumor tissue, indicating that the inulin hydrogel matrix in Oxa@HMI hydrogel can be degraded by probiotics within tumor tissue to produce butyric acid, which induced CD8^+^ T cells to express more ID2 and IL-12 receptors for immune response activation. As shown in Fig. 8C to F, the inulin-presented groups (inulin gel and Oxa@HMI hydrogel groups) exhibited significantly higher levels of tumor-infiltrating CD8^+^ T and CD4^+^ T cells compared to the PBS group detected by flow cytometry and immunofluorescence staining assays. It is worth noting that Oxa is a highly immunogenic chemotherapeutic drug that triggers the antitumor immune responses by inducing immunogenic cell death (ICD) effects. Therefore, both mono- or combined therapy by Oxa and inulin increased the infiltration levels of CD8^+^ T and CD4^+^ T cells. Similarly, as an important cytokine secreted by CD8^+^ T cells, IFN-γ in serum showed significant upregulation, further demonstrating the promotion of effector T cell infiltration and function by Oxa@HMI hydrogel (Fig. 8G).

**Fig. 9.**
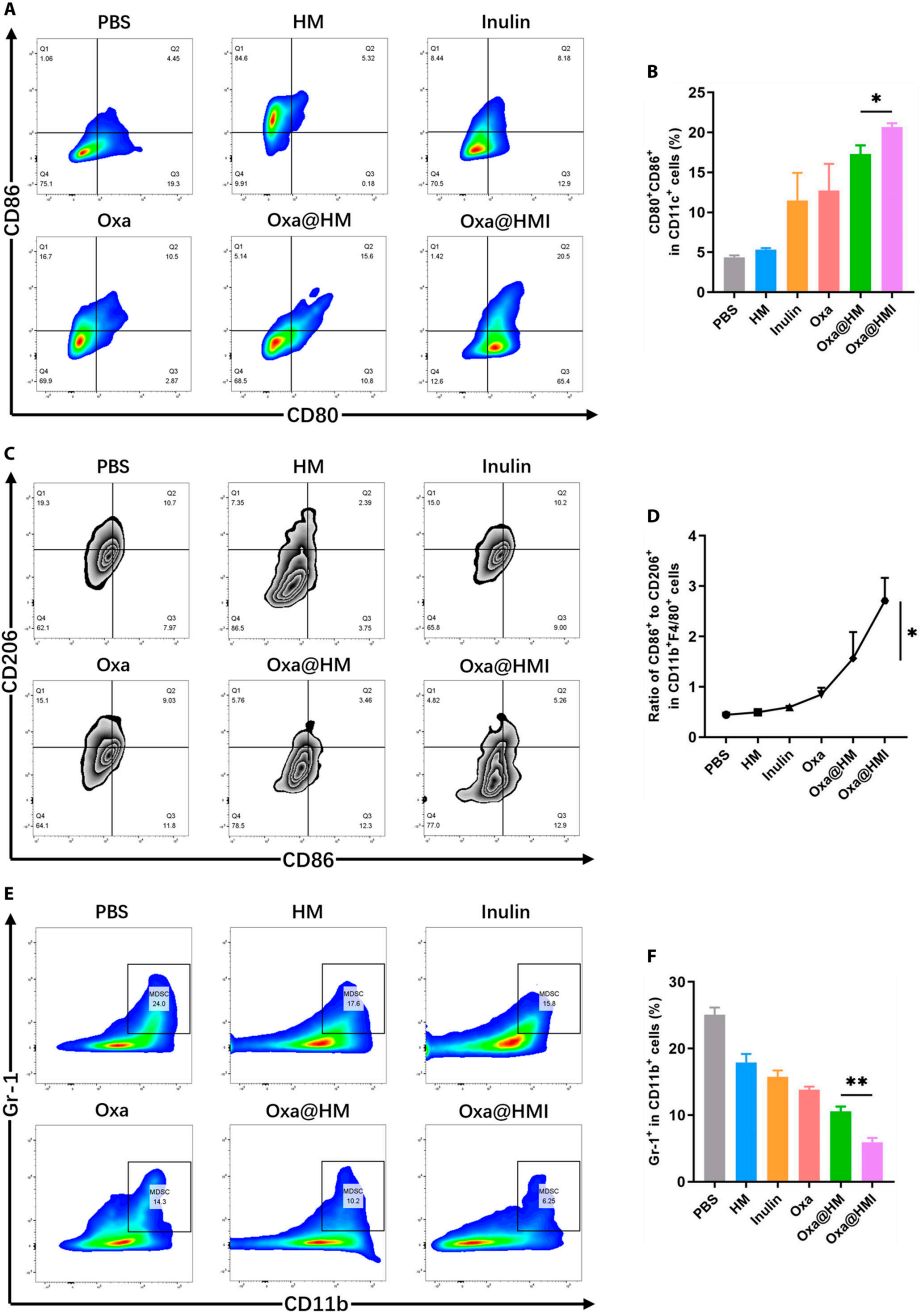
The tumor immune microenvironment modulation by Oxa@HMI Gel via regulating intratumoral microbiota on orthotopic colorectal tumor-bearing Balb/c mice. Representative flow cytometric images (A) and the corresponding quantitative analysis (B) of DCs (CD80^+^CD86^+^ gated by CD11c^+^) in TME after diverse treatments (*n* = 3). Representative flow cytometric images (C) and the corresponding quantitative analysis (D) of the ratio of M1 (CD86^+^) to M2 (CD206^+^) macrophages (gated by CD11b^+^F4/80^+^) in TME after various treatments (*n* = 3). Representative flow cytometric images (E) and the corresponding quantitative analysis (F) of MDSCs (Gr-1^+^ gated by CD11b^+^) infiltrated in TME after various treatments (*n* = 3). The data are presented as the mean ± SD. *P < 0.05, **P < 0.01.

To comprehensively understand the relationship between microbiota regulation and immunity, we also conducted evaluation of other relevant immune cells within the TME. Oxa@HMI hydrogel effectively induced DCs maturation (CD80^+^CD86^+^), indicating a significant activation of antigen-presenting function (Fig. 9A and B). In addition, Oxa@HMI hydrogel significantly promoted the polarization of tumor-associated M2 macrophages to proinflammatory M1 macrophages, with an M1/M2 ratio nearly twice that of the Oxa@HM NPs and the Oxa groups (Fig. 9C and D). Moreover, compared with other groups, the Oxa@HMI hydrogel group showed the lowest level of immunosuppressive MDSCs in tumor tissue (Fig. 9E and F). These effects can be attributed to the SCFAs generated by the fermentation degradation of the inulin hydrogel matrix in Oxa@HMI hydrogel by the microbiota, which bind to GPR43 as well as to induce IL-12 receptor expression on the surface of CD8^+^ T cells, while the presence of Oxa in the prescription synergistically enhanced this immune activation effect.

### Antitumor efficacy of Oxa@HMI hydrogel on subcutaneous colorectal tumor-bearing mice

**Fig. 10.**
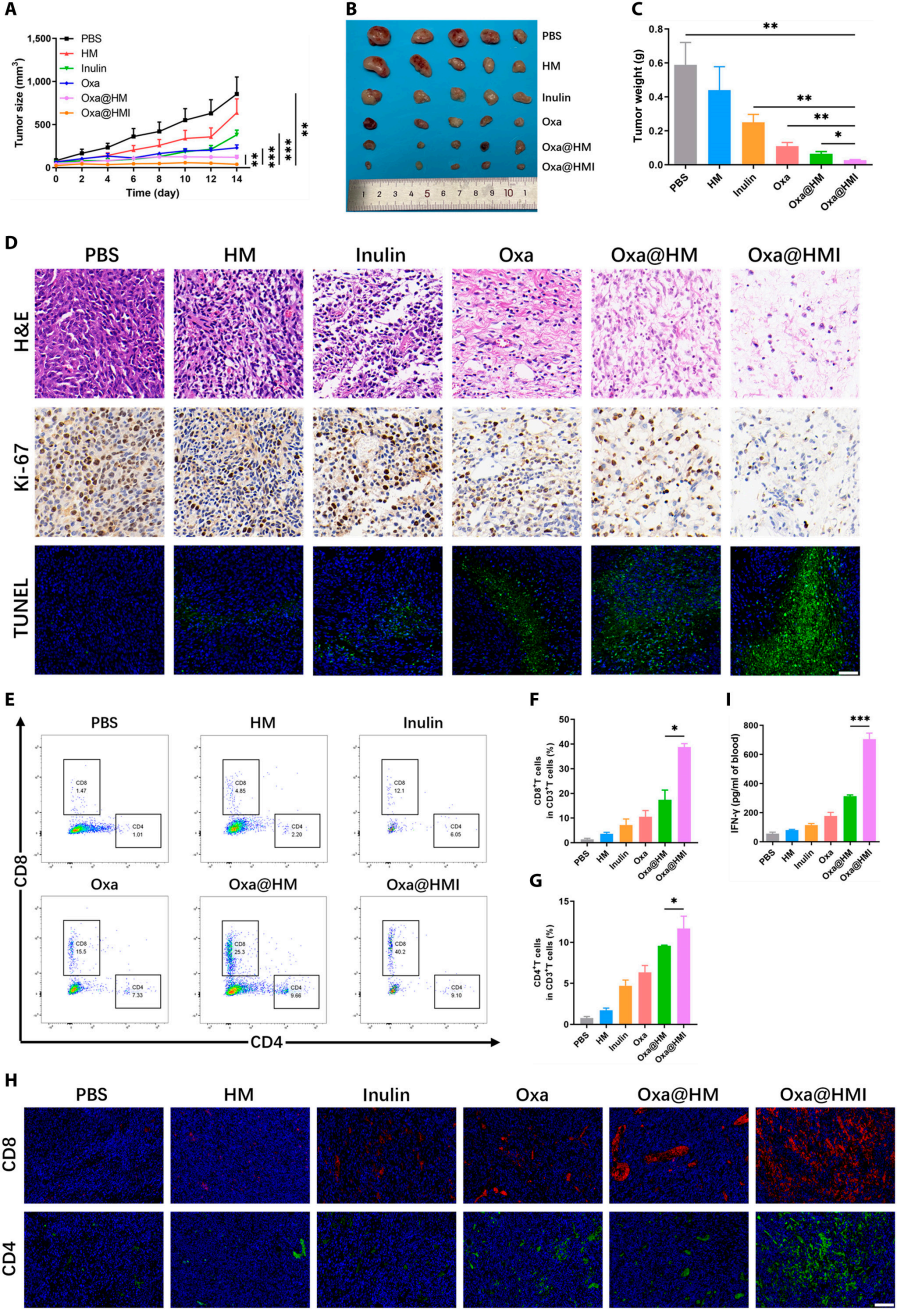
In vivo antitumor activity and the tumor immune microenvironment modulation by Oxa@HMI Gel on subcutaneous colorectal tumor-bearing Balb/c mice. (A) The tumor growth curves of subcutaneous colorectal tumor-bearing mice received various treatments (*n* = 5). Representative images (B) and weight (C) of tumors excised from the sacrificed mice after medication process (*n* = 5). (D) Histological analysis by H&E, Ki-67, and TUNEL staining of tumor sections after various treatments. Scale bar: 20 μm. Representative flow cytometric images (E) and corresponding quantitative analysis of CD8^+^ T cells (F) and CD4^+^ T cells (G) infiltrated in the tumor tissues form mice after different treatments (*n* = 3). (H) Immunofluorescent staining images of CD8^+^ T cells and CD4^+^ T cells in tumor tissues from mice after various treatments. Scale bar: 20 μm. (I) The secretion levels of serum IFN-γ determined by enzyme-linked immunosorbent assay (*n* = 3). The data are presented as mean ± SD. *P < 0.05, **P < 0.01, ***P < 0.001.

In order to further explore the in vivo antitumor mechanism of Oxa@HMI hydrogel, we established a subcutaneous colorectal cancer model using CT26 cells. When the tumor reached a volume of 100 mm^3^, different formulations were orally administered for tumor therapy. As shown in Fig. 10A, both inulin gel and free Oxa groups showed limited antitumor effects. Meanwhile, the Oxa@HM NPs group, possibly due to the presence of MnO_2_, which may have altered the anaerobic ecological environment of the intestinal microbiota, promoted the inhibitory effect of Oxa on tumor growth. More importantly, the Oxa@HMI hydrogel almost completely suppressed subcutaneous tumor growth at the end of treatment, with significantly lower tumor volume and weight than the other groups (Fig. 10B and C). Histological examination also confirmed evident apoptotic markers and negligible proliferation markers in tumor tissue (Fig. 10D).

By investigating the levels of CD8^+^ T and CD4^+^ T cells infiltration in tumor tissue through flow cytometry and immunofluorescence staining, it was found that monotherapy by inulin or Oxa could exert certain immune activation effects. Moreover, in the presence of both components, the Oxa@HMI hydrogel treatment achieved a synergistic immunotherapeutic effect (Fig. 10E to I). These results were consistent with the findings in the orthotopic colorectal cancer model, indicating that oral administration of Oxa@HMI hydrogel exerted a series of biofunctional effects, including prolonged intestinal retention, microbiota regulation, and immune activation, which effectively inhibited the growth of orthotopic colorectal cancer, as well as subcutaneous tumor through activating systemic immune responses.

### In vivo biosafety

The biosafety of nanomedicines during sustained administration is also a critical issue. During the treatment period, mice in both orthotopic and subcutaneous tumor groups showed a slow and steady increase in body weight (Fig. 5E and Fig. S6). Serum biochemical parameters related to liver (aspartate aminotransferase AST; alanine aminotransferase, ALT), kidney (creatinine, Cr; blood urea nitrogen, BUN), and heart (creatine kinase, CK) functions in subcutaneous tumor-bearing mice receiving various treatments showed no significant abnormalities (Fig. S7A to E). Furthermore, H&E staining of major organs from mice in each group showed no apparent damage (Fig. S8). In summary, as a colon-targeted oral administration system for tumor therapy, Oxa@HMI hydrogel is safe, effective, and free from toxic side effects.

## Discussion

In this study, we have designed an orally administered hydrogel, Oxa@HMI, effectively promoted antitumor immune responses and enhanced chemotherapy efficacy by modulating the microbiota microenvironment on both subcutaneous and orthotopic colorectal cancer mouse models. The observed satisfactory antitumor effects of Oxa@HMI hydrogel can be attributed to the following mechanisms: (a) The inulin gelatinous shell protected the drug from the acidic and enzymatic environment in the upper gastrointestinal tract and was selectively degraded by beneficial bacteria in the colon, thus facilitating efficient colon-targeted drug release. (b) The degradation of the outer inulin layer generated SCFAs, which not only modulated the microbiota microenvironment but also triggerred a series of cascade antitumor immune responses. (c) After degradation, the internal nanomedicine Oxa@HM was exposed to generate O_2_ and ROS in response to the acidic TME, synergistically exerting tumor-targeted cytotoxicity with the chemotherapeutic drug Oxa. In summary, the designed orally administered hydrogel Oxa@HMI exhibited specific regulation of the intestinal microbiota and tumor microbiota, thereby activating a series of antitumor immune responses and enhancing chemotherapy effects.

## Materials and Methods

### Materials

Inulin, anhydrous sodium carbonate, 3-aminopropyltriethoxysilane, and tetraethyl orthosilicate (TEOS) were received from Aladdin (Shanghai, China). Oxaliplatin, Triton X-100, KMnO_4_, cyclohexane, n-hexanol, ammonia water, and hydrogen peroxide (w/w: 30%) were received from Macklin (Shanghai, China). ROS detection fluorescent probe dichlorofluorescein diacetate (DCFH-DA) and O_2_ indicator probe ([Ru(dpp)_3_] Cl_2_) were received from Biyun Tian (Shanghai, China). ICG and D-lucifer salt were received from Yisheng (Shanghai, China). SCF, SIF, SGF, and inulinase were received from Yuan Ye (Shanghai, China). Trypsin, RPMI 1640 medium, PBS, and fetal bovine serum were received from Inner Mongolia Opcel Biotechnology Co., Ltd. All reagents used in this work were analytical grade and used without any further purification.

### Characterization

The morphology and structure of Oxa@HMI NPs and Oxa@HMI hydrogel were observed using transmission electron microscope (JEOL-2100) and scanning electron microscope (Zeiss SIGMA). The main element distribution of Oxa@HMI hydrogel was studied using high-angle annular dark-field scanning TEM (Oxford x-met 8000). The UV-vis spectrum was measured on a UV-3600 UV-vis spectrophotometer (Lambda Bio40, PerkinElmer). Drug loading was measured by ICP-MS analysis. The zeta potential, PDI, and particle size of the NPs were measured on a Malvern Zetasizer (Nano-ZS ZEM3600).

### Preparation of HM NPs

A mixture of 10.6 ml of Triton X-100, 45 ml of cyclohexane, and 10.8 ml of n-hexanol was stirred for 5 min. Then, 1.5 ml of ammonia water and 2 ml of deionized water were added and stirred for 30 min. Subsequently, 1 ml of TEOS and 0.2 ml of 3-aminopropyltriethoxysilane were added and stirred for 24 h. After centrifugation for 20 min, the obtained precipitation was washed with ethanol and deionized water 3 times to obtain sSiO_2_.

Subsequently, 100 mg of sSiO_2_ was ultrasonicated in 100 ml of deionized water to achieve dissolution. The KMnO_4_ solution was then gradually added to the sSiO_2_ solution and stirred for an extended period overnight, resulting in the formation of MnO_2_@sSiO_2_.

Finally, 21.198 g of anhydrous NaCO_3_ was dissolved in 80 ml of deionized water, then MnO2@sSiO2 solution was added to the NaCO_3_ solution and sonicated slightly to ensure uniformity. The mixture was then heated in a 60 °C oil bath for 12 h, followed by centrifugation at 12,500 rpm and washing with deionized water to obtain HM NPs.

### Preparation of Oxa@HMI NPs and Oxa@HMI hydrogel

10 ml of HM NPs solution (2 mg/ml) was mixed with 10 ml of Oxa solution (4 mg/ml) and subjected to stirring for a 24-h period. This process yielded Oxa-loaded Oxa@HM NPs. Then, 0.4 g of inulin was dissolved into 4 ml of Oxa@HM NPs solution, followed by centrifugation to remove the unreacted inulin, we can obtain Oxa@HMI NPs.

Similarly, 1.6 g of inulin was dissolved in 4 ml of Oxa@HM NPs solution, stirring in a 70 °C water bath for 6 h until all the inulin was dissolved. Subsequently, the mixed solution was placed in a refrigerator at a temperature of 4 °C and left overnight to facilitate the formation of Oxa@HMI hydrogel.

### Stability of Oxa@HMI NPs in simulated gastrointestinal fluids

The physical stability of NPs was evaluated in simulated gastrointestinal fluids, including SGF (pH 1.2), SIF (pH 7.4), and SCF (pH 6.8). One milliliter of Oxa@HMI NPs solution was added into 9 ml of simulated gastrointestinal fluid and incubated for a specific period of time (2 h for SGF group, 4 h for SIF group, and 8 h for SCF group). The average particle size (nm), PDI, and zeta potential (mV) of Oxa@HMI NPs were measured and assessed, based on the initial measurements before incubation [71,72].

### Drug release studies

To evaluate the release of Oxa, Oxa@HMI NPs (coincubation with inulinase, 10 U/ml) were oscillated and dialyzed in PBS solutions of different pH values (5.5, 6.5, and 7.4) at 37 °C for 24 h. At specific time intervals, 1 ml of the release medium was removed and replaced with 1 ml of fresh medium. The amount of released Oxa at different time points was determined using ICP-MS analysis.

### Cell culture

Mouse colon cancer CT26 and the luciferase-labeled CT26-Luc cell lines were acquired from American Type Culture Collection. These tumor cells were cultured in RMPI-1640 medium containing 10% fetal bovine serum and 1% antibiotics at 37 °C in an atmosphere of 5% CO_2_.

### Cellular uptake

CT26 cells were seeded into culture dishes and incubated for 24 h. Cells were then coincubated with fluorescent probe RB-labeled NPs, including RB@HM NPs, RB@HMI NPs, and RB@HMI NPs (coincubated with inulinase, 10 U/ml), for 2, 4, and 8 h, respectively. Subsequently, flow cytometry was employed to determine the cellular uptake of various NPs by CT26 cells at different time points.

### Cytotoxicity evaluation

Cell viability of CT26 cells was measured using MTT assay, live/dead staining, and Annexin V-fluorescein isothiocyanate (FITC) and propidium iodide (PI) staining, respectively. Briefly, cells were seeded in a culture plate for 24 h and then treated with various solutions with Oxa at different concentrations (coincubation with inulinase, 10 U/ml). After 24 h of treatment, cells were collected for MTT kit detection. Cells were also stained using the live/dead assay kit (Calcein-AM indicates live cells, green fluorescence; PI indicates dead cells, red fluorescence) for fluorescence microscope observation. Annexin V-FITC and PI staining were used to detect the cell apoptosis of CT26 cells, and apoptosis levels were analyzed using flow cytometry.

### Determination of generated O_2_ level in vitro

Different NP-containing solutions (coincubatiom with inulinase, 10 U/ml) were added into nitrogen saturated water pretreated with H_2_O_2_ (100 μm). The dissolved O_2_ levels in the liquid at a paraffin-sealed environment were determined using an O_2_ meter.

The intracellular generation of O_2_ was assessed using an O_2_ probe [Ru(dpp)_3_]Cl_2_. Briefly, CT26 cells were seeded in a 24-well plate and incubated with different formulations (coincubation with inulinase, 10 U/ml) under hypoxic conditions for 24 h (Oxa concentration 50 μg/ml), and then were incubated with [Ru(dpp)_3_]Cl_2_ at a concentration of 10 μg/ml for 12 h. The intracellular O_2_ content was assessed by detecting the fluorescence of intracellular [Ru(dpp)_3_]^2+^ by confocal laser scanning microscope (CLSM) observation.

### Determination of ROS level in vitro

The ROS level in solution was determined through the decreased UV-vis absorbance of methylene blue in presence of ROS. In brief, PBS, Oxa, HM, Oxa@HM, Oxa@HMI, and Oxa@HMI (coincubation with inulinase, 10 U/ml) were dissolved in water containing H_2_O_2_ (100 μm). Subsequently, 0.01% methylene blue was added to the solution. The absorbance of the resulting solution was measured at 664 nm.

The intracellular ROS level was determined using DCFH-DA staining assay. The CT26 cells were seeded in a 24-well plate for 24 h and then incubated with different NPs (coincubation with inulinase, 10 U/ml) for another 24 h (Oxa 50 μg/ml). After that, the cells were stained with DCFH-DA (10 μM), and the intracellular ROS level was assessed by detecting the fluorescence of DCF through CLSM observation.

### Animal tumor models

Female Balb/c mice (6 to 8 wk old) were obtained from the Experimental Animal Center of Southern Medical University. All animal studies were approved by the Animal Ethics Committee of Southern Medical University (No. SMUL2022187). Subcutaneous colon tumor models were established by injecting 2 × 10^6^ CT26 cells (suspended in 100 μl of PBS) subcutaneously into the dorsal region of the mice. Orthotopic colon tumor models were established by injecting 3 × 10^6^ C26-Luc tumor cells (suspended in 50 μl of PBS) into the submucosal layer of the ascending colon wall. Tumor growth in the orthotopic and subcutaneous models was monitored using an IVIS (PerkinElmer) and tumor volume measurement (Width^2^ × Length/2) with a digital measurement caliper, respectively.

### Biodistribution

The orthotopic colorectal tumor-bearing mice were gavaged with ICG, ICG@HM NPs, ICG@HMI NPs, and ICG@HMI hydrogel, respectively (ICG 20 mg/kg). Each mouse was photographed using an IVIS at different time points (2, 4, 6, 8, 12, and 24 h). At 24 h, the mice were euthanized, and the colon and colon tumors were dissected for ex vivo ICG fluorescence detection to assess the retention and distribution of the orally administrated formulations.

### In vivo antitumor effect

Both orthotopic CT26-Luc and subcutaneous CT26 tumor-bearing mice were randomly divided into 6 groups (*n* = 5). When the tumor volume reached approximately 100 mm^3^, mice were orally administrated with different formulations (PBS, HM NPs, free Oxa, inulin Gel, Oxa@HM NPs, and Oxa@HMI hydrogel) once a day for a total of 14 d (Oxa 20 mg/kg). Tumor size and body weight of the mice were monitored every 2 d during the administration period. After the medication process, all tumor-bearing mice were sacrificed, and tumors as well as major organs from each group were gathered and fixed with 4% paraformaldehyde for subsequent TUNEL and H&E staining assays.

### Flow cytometry analysis

The FACSCanto II flow cytometer (BD Biosciences) was used to detect immune cells in tumor tissues. Single-cell suspensions of tumors were prepared according to the method described by Weigmann *et al.* [73]. To analyze CD8^+^ T cells (CD8^+^CD3^+^) and CD4^+^ T cells (CD4^+^CD3^+^), cells were stained with anti-CD3-APC/Cyanine7, anti-CD4-FITC, and anti-CD8-PE antibodies (Biolegend) following the manufacturer’s instructions. For macrophage polarization, cells were stained with anti-CD206-FITC, anti-CD11b-PE, anti-CD86-PE/Cyanine7, and anti-F4/80->Alexafluor 647 antibodies (Biolegend). CD11b^+^F4/80^+^CD86^+^ and CD11b^+^F4/80^+^CD206^+^ cells were defined as M1 and M2 macrophages, respectively. To analyze DC cells (CD11c^+^CD80^+^CD86^+^), cells were stained with anti-CD11c-FITC, anti-CD80-PE, and anti-CD86-PE/Cyanine7 antibodies (Biolegend). To analyze MDSCs (CD11b^+^Gr-1^+^), cells were stained with anti-CD11b-PE/Cyanine7 and anti-Gr-1-APC antibodies (Biolegend). Flow cytometry data analysis was conducted using FlowJo 10.8.1 software.

### Biosafety

Blood samples were gathered and centrifuged (4 °C, 3,000 rpm, 10 min) to separate serum after the treatment. The systemic toxicity of oral treatment with Oxa@HMI hydrogel was assessed by measuring the serum levels of alanine aminotransferase (ALT), aspartate aminotransferase (AST), creatinine (Cr), blood urea nitrogen (BUN), and creatine kinase (CK) in tumor-bearing mice.

### Statistical analysis

All values were expressed as mean ± standard deviation (SD), and all experiments were performed 3 times unless otherwise noted. Statistical analysis was performed using an unpaired *t* test and 1-way analysis of variance (ANOVA) with multiple comparisons. Statistical significance was denoted as follows: *P < 0.05, **P < 0.01, ***P < 0.001, and ****P < 0.0001. The Prism 8.0 software (GraphPad Software) was used for statistical analysis.

## Data Availability

The data generated in this study are available in the article and supplementary data or upon request from the corresponding author.
