# Supplementary material for: Orally Administrated Hydrogel Harnessing Intratumoral Microbiome and Microbiota-Related Immune Responses for Potentiated Colorectal Cancer Treatment
Source: Research (Wash D C). 2024 May 8;7:0364. doi: 10.34133/research.0364 (PMC11077293; doi:10.34133/research.0364)
Supplement: Supplementary 1 — Figs. S1 to S18 [file research.0364.f1.docx]

**Supplementary Material**


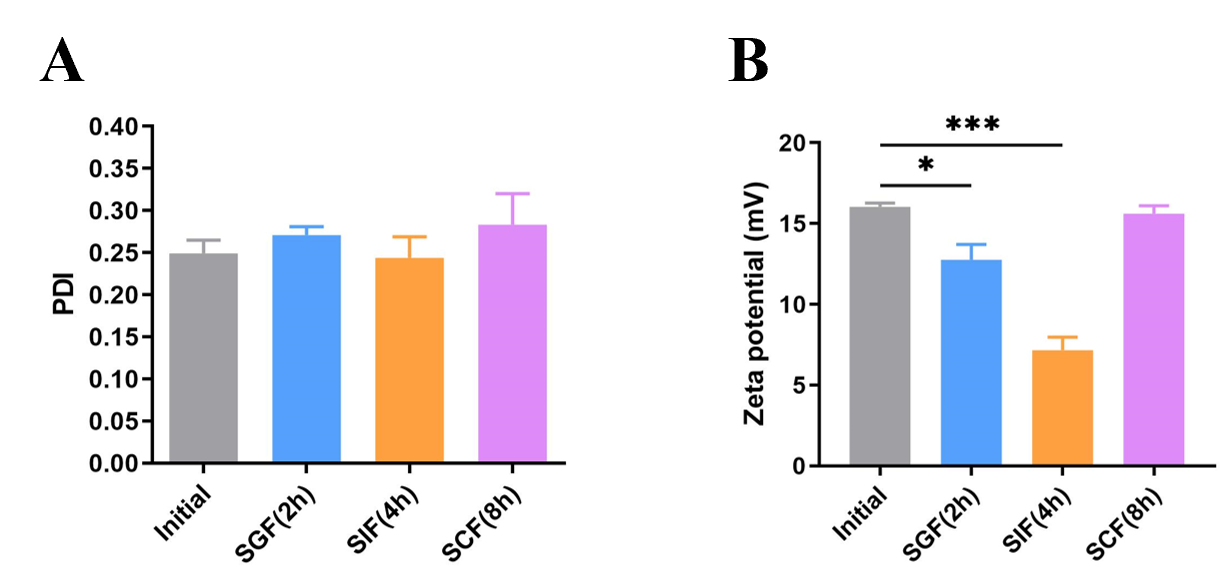


**Fig. S1.** PDI (A) and Zeta potential (B) of Oxa@HMI NPs after exposure to Simulated Gastric Fluid (SGF), Simulated Intestinal Fluid (SIF), Simulated Colonic Fluid (SCF) for 2h, 4h, and 8h, respectively (n = 3). The data are presented as the mean ± SD. *P < 0.05, ***P < 0.001.


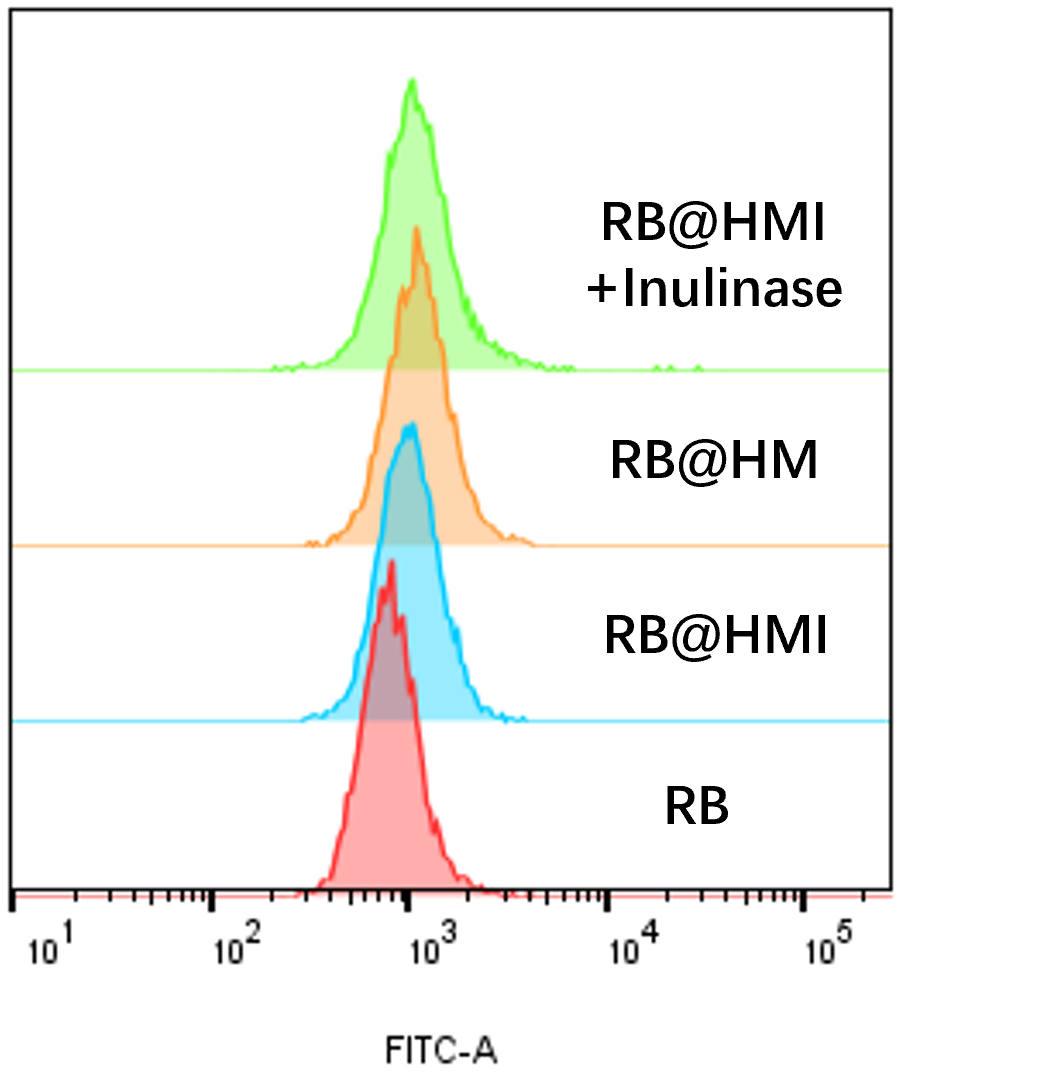


**Fig. S2.** Cellular uptake analysis in CT26 cells analyzed by flow cytometry after treatment with RB, RB@HMI NPs, RB/HM NPs, RB@HMI NPs with inulinase for 8 h, respectively.


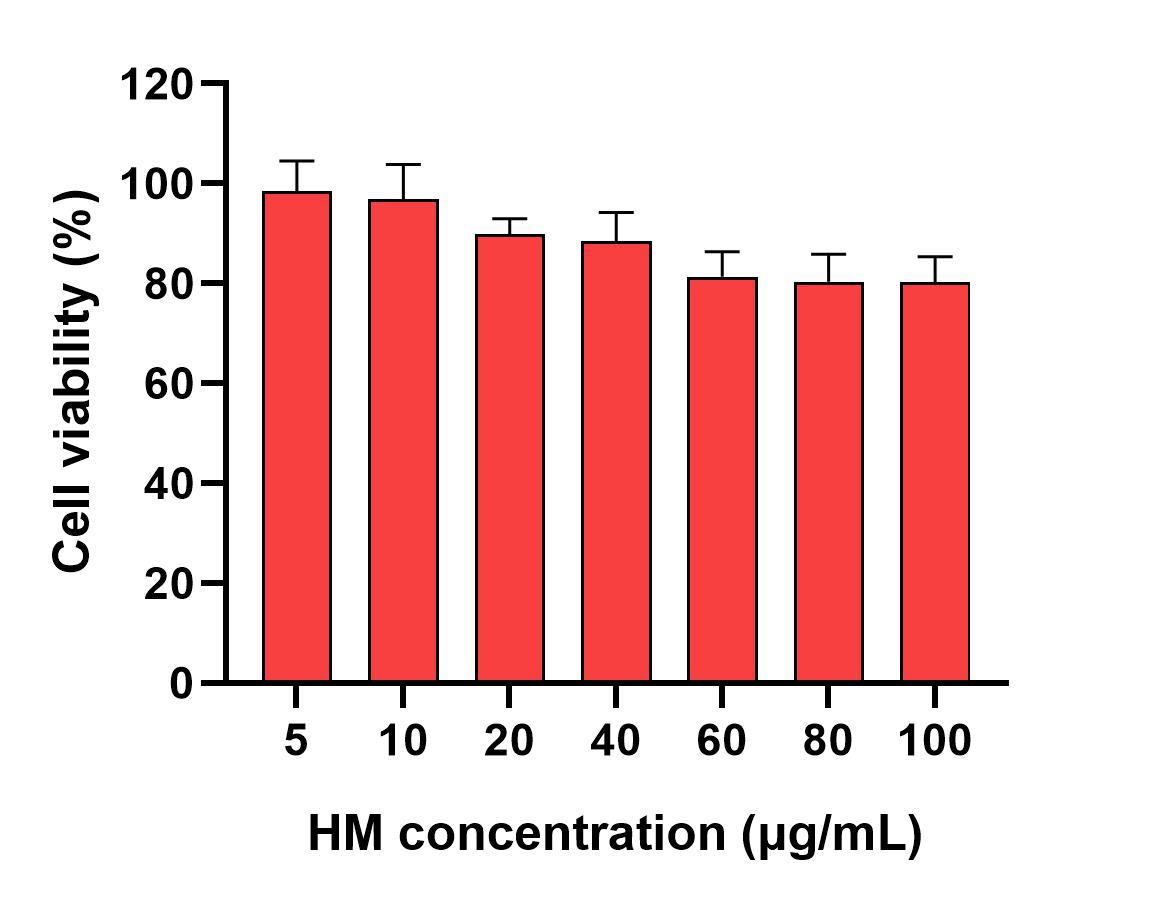


**Fig. S3.** Cell viabilities of CT26 cells after being incubated with various concentrations of HM for 24 h (n = 5). The data are presented as the mean ± SD.


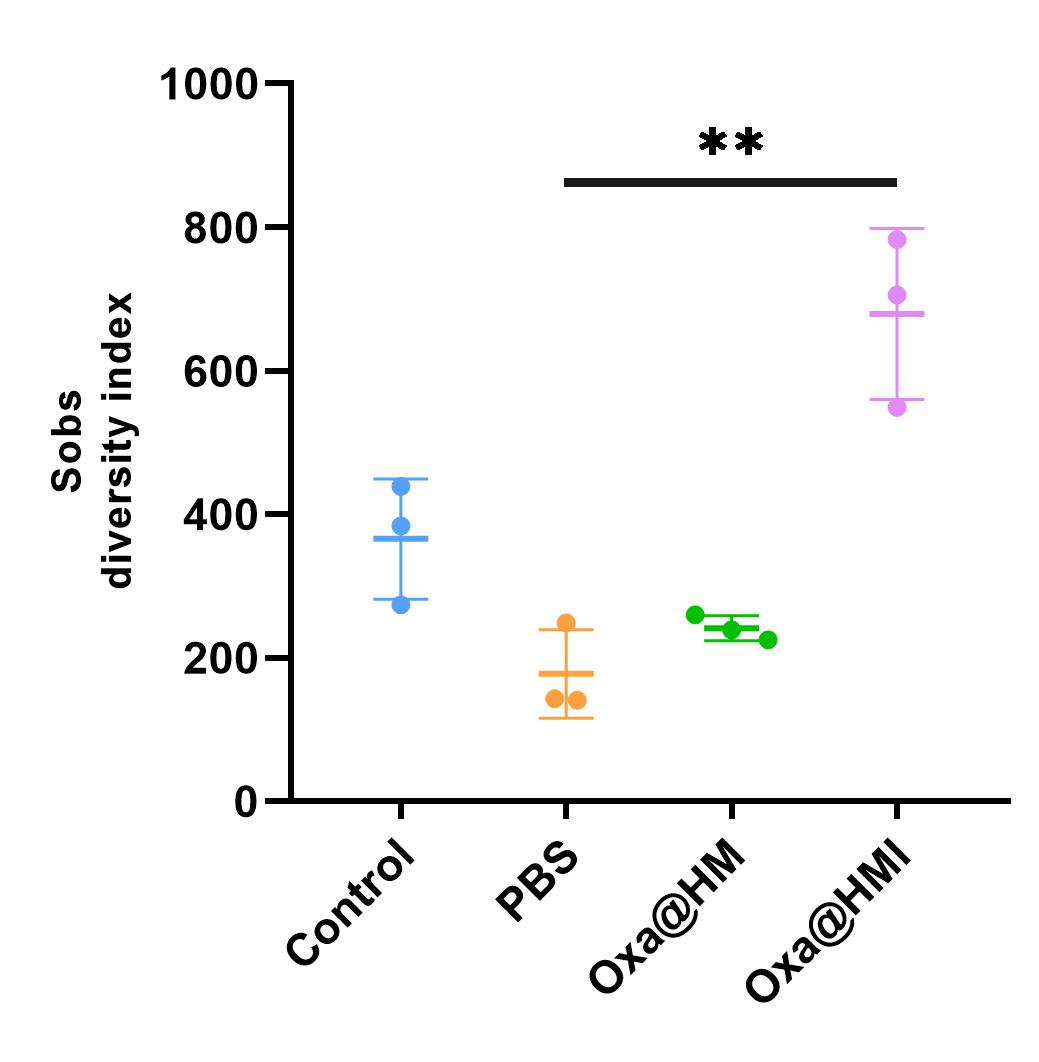


**Fig. S4.** The richness analysis of gut microbiota from various treated mice expressed as Sobs index.


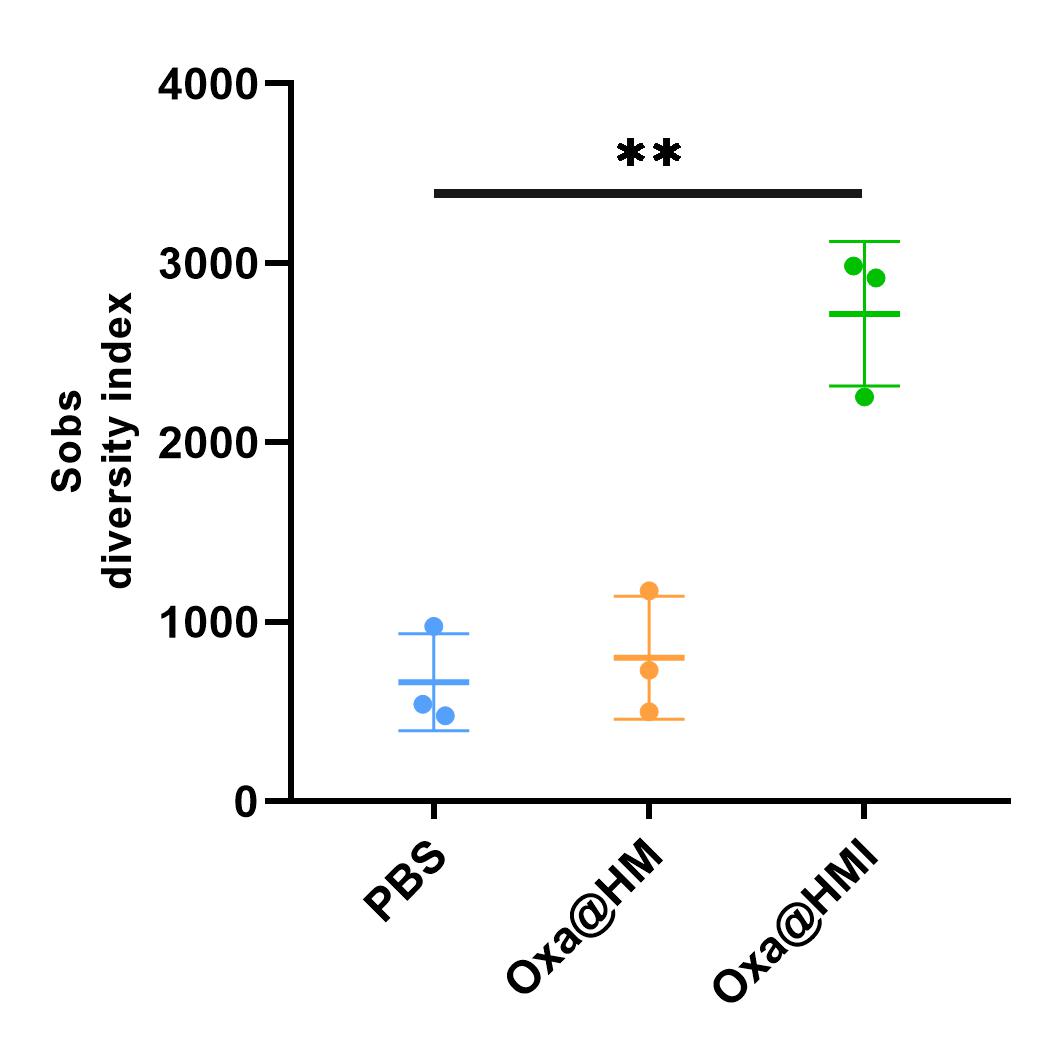


**Fig. S5.** The richness analysis of intratumoral microbiota from various treated mice expressed as Sobs index.


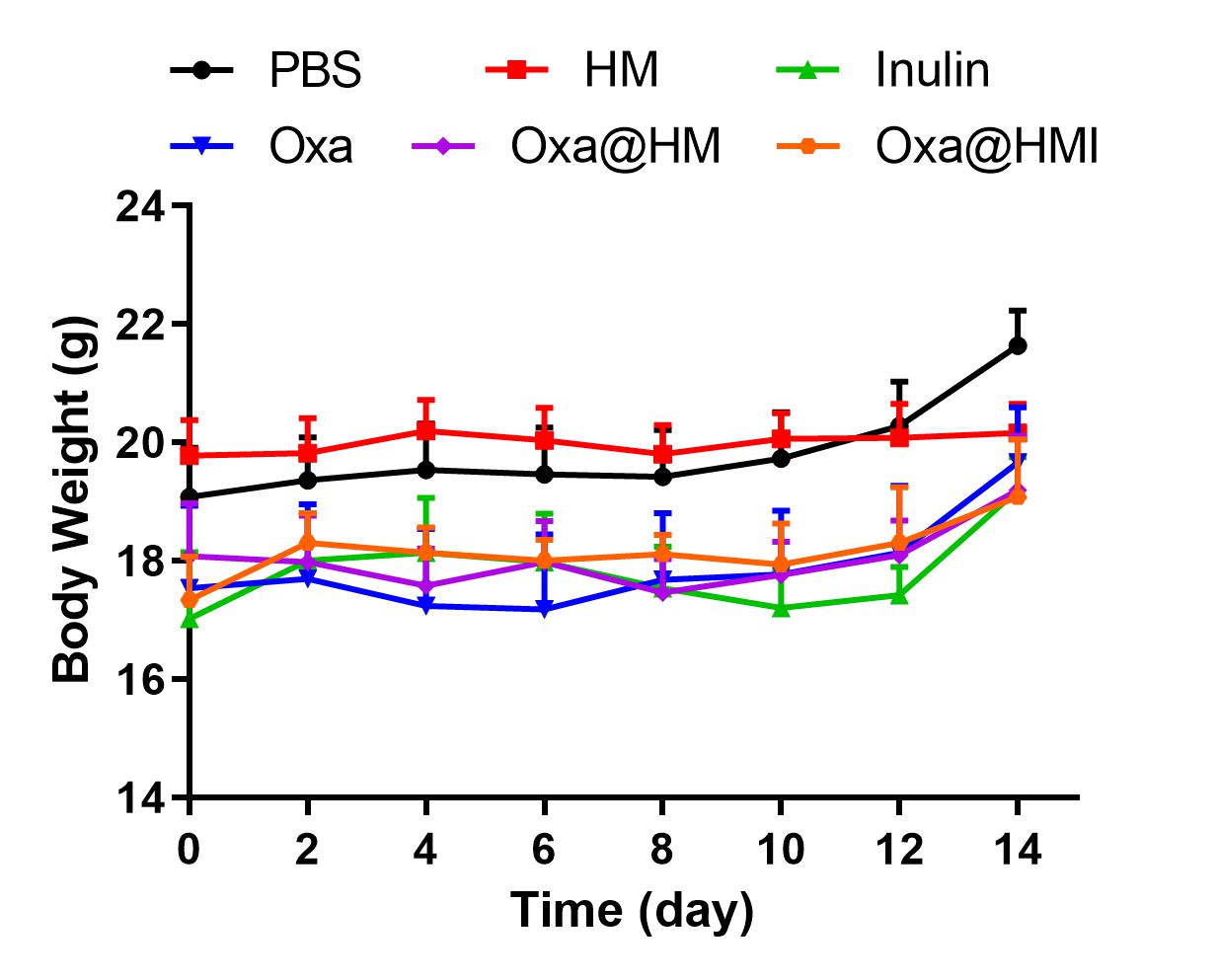


**Fig. S6.** Body weight variation of subcutaneous colorectal tumor-bearing mice receiving various treatments (n = 5). The data are presented as the mean ± SD.


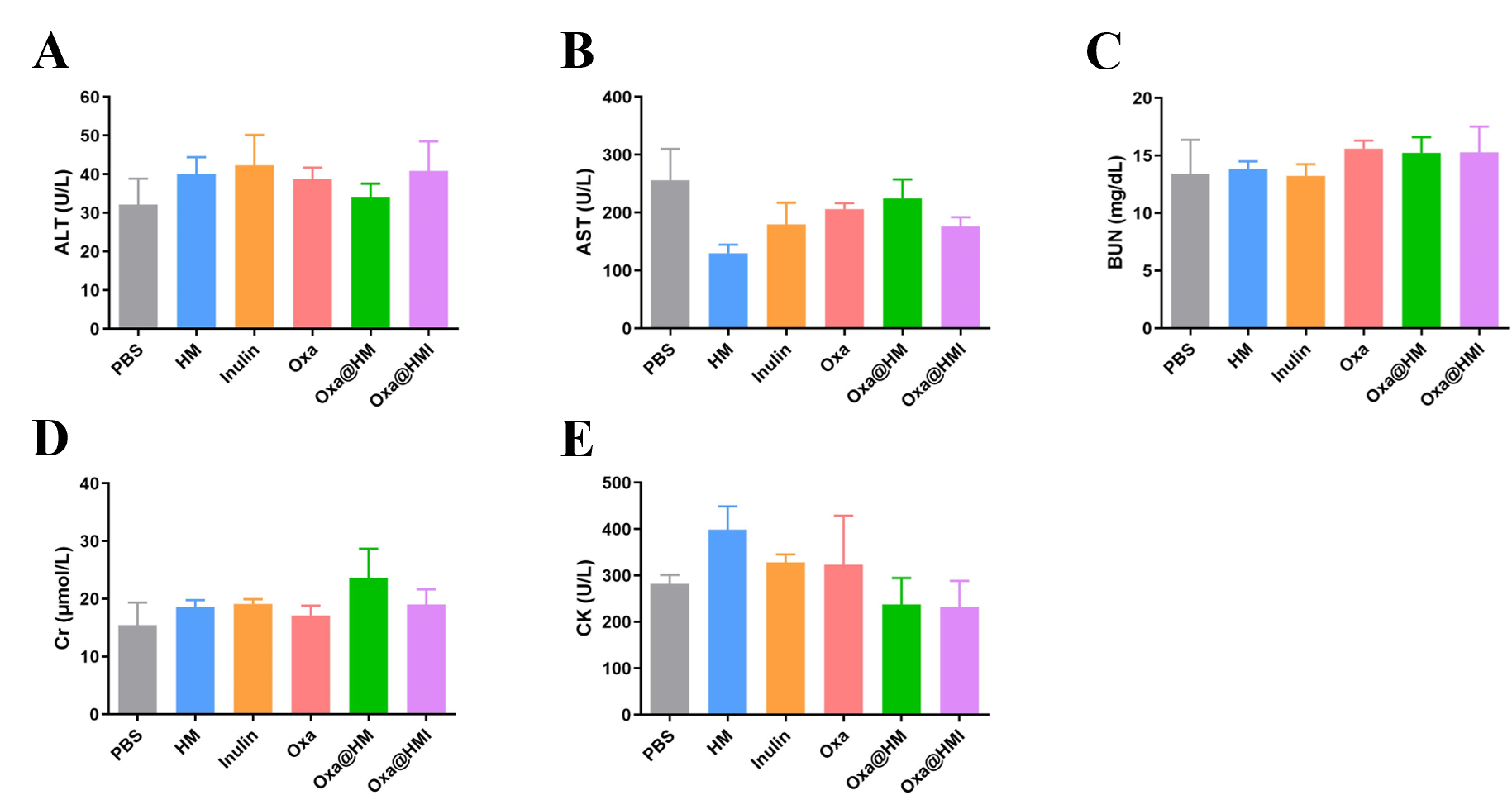


**Fig. S7.** The safety evaluation assessed by the concentration of serum biochemical indicators, including ALT (A), AST (B), BUN (C), Cr (D), and CK (E) of subcutaneous colorectal tumor-bearing mice after various treatments (n = 3). The data are presented as the mean ± SD.


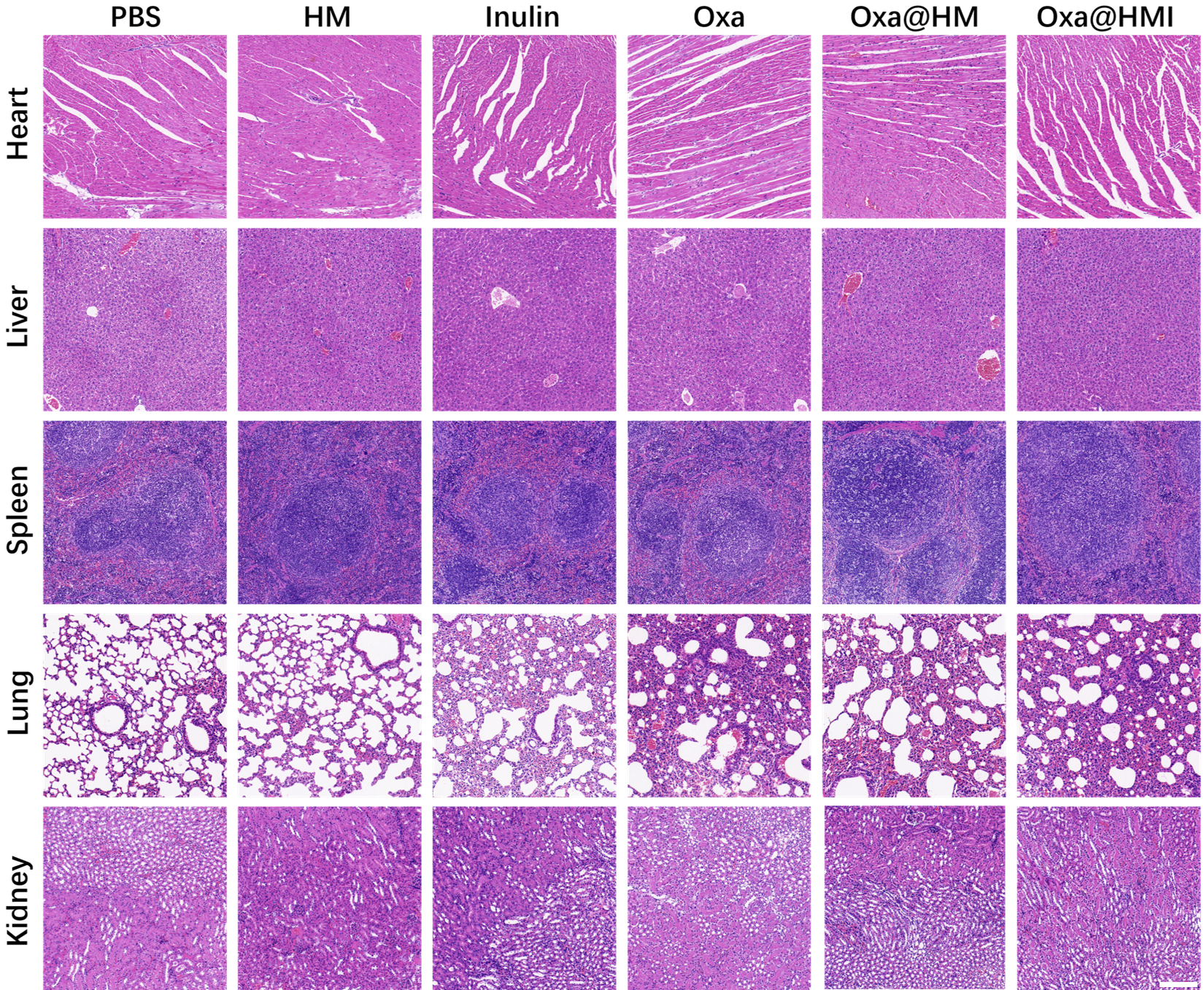


**Fig. S8.** H&E staining sections of major organs (Heart, Liver, Spleen, Lung, Kidney) after various treatments (subcutaneous colorectal tumor-bearing mice). Sacle bar: 50 μm.
